# Single-cell RNA-seq reveals alterations in peripheral *CX3CR1* and nonclassical monocytes in familial tauopathy

**DOI:** 10.1186/s13073-023-01205-3

**Published:** 2023-07-18

**Authors:** Daniel W. Sirkis, Caroline Warly Solsberg, Taylor P. Johnson, Luke W. Bonham, Virginia E. Sturm, Suzee E. Lee, Katherine P. Rankin, Howard J. Rosen, Adam L. Boxer, William W. Seeley, Bruce L. Miller, Ethan G. Geier, Jennifer S. Yokoyama

**Affiliations:** 1 Memory and Aging Center, Department of Neurology, Weill Institute for Neurosciences, University of California, San Francisco, 1651 4th Street, San Francisco, CA 94158 USA; 2 Pharmaceutical Sciences and Pharmacogenomics Graduate Program, University of California, San Francisco, CA 94158 USA; 3 Department of Radiology and Biomedical Imaging, University of California, San Francisco, CA 94158 USA; 4 Global Brain Health Institute, University of California, San Francisco, CA 94158 USA; 5 Trinity College Dublin, Dublin, Ireland; 6 Department of Pathology, University of California, San Francisco, CA 94158 USA; 7 Transposon Therapeutics, Inc, San Diego, CA 92122 USA

**Keywords:** Tau, Tauopathy, MAPT, CX3CR1, Dementia, Neurodegeneration, Nonclassical monocytes, Microglia, PBMCs, Single-cell RNA-seq

## Abstract

**Background:**

Emerging evidence from mouse models is beginning to elucidate the brain’s immune response to tau pathology, but little is known about the nature of this response in humans. In addition, it remains unclear to what extent tau pathology and the local inflammatory response within the brain influence the broader immune system.

**Methods:**

To address these questions, we performed single-cell RNA sequencing (scRNA-seq) of peripheral blood mononuclear cells (PBMCs) from carriers of pathogenic variants in *MAPT*, the gene encoding tau (*n* = 8), and healthy non-carrier controls (*n* = 8). Primary findings from our scRNA-seq analyses were confirmed and extended via flow cytometry, droplet digital (dd)PCR, and secondary analyses of publicly available transcriptomics datasets.

**Results:**

Analysis of ~ 181,000 individual PBMC transcriptomes demonstrated striking differential expression in monocytes and natural killer (NK) cells in *MAPT* pathogenic variant carriers. In particular, we observed a marked reduction in the expression of *CX3CR1*—the gene encoding the fractalkine receptor that is known to modulate tau pathology in mouse models—in monocytes and NK cells. We also observed a significant reduction in the abundance of nonclassical monocytes and dysregulated expression of nonclassical monocyte marker genes, including *FCGR3A*. Finally, we identified reductions in *TMEM176A* and *TMEM176B*, genes thought to be involved in the inflammatory response in human microglia but with unclear function in peripheral monocytes. We confirmed the reduction in nonclassical monocytes by flow cytometry and the differential expression of select biologically relevant genes dysregulated in our scRNA-seq data using ddPCR.

**Conclusions:**

Our results suggest that human peripheral immune cell expression and abundance are modulated by tau-associated pathophysiologic changes. *CX3CR1* and nonclassical monocytes in particular will be a focus of future work exploring the role of these peripheral signals in additional tau-associated neurodegenerative diseases.

**Supplementary Information:**

The online version contains supplementary material available at 10.1186/s13073-023-01205-3.

## Background

Nearly 25 years after the discovery of pathogenic variants in *MAPT* (encoding the microtubule-associated protein tau) in familial frontotemporal dementia (FTD; [1, 2]; reviewed in [3]), there are still no effective therapeutics capable of halting or delaying tau-associated neurodegeneration [4, 5]. Diverse tau proteinopathies (tauopathies) also occur sporadically (i.e., in the absence of *MAPT* or other pathogenic variants) and are subdivided into primary tauopathies—which collectively fall under the umbrella term, frontotemporal lobar degeneration (FTLD)-tau—and secondary tauopathies, the most prominent example of which is Alzheimer’s disease (AD). Although much effort has gone into characterizing the natural history and longitudinal declines of *MAPT* pathogenic variant carriers (reviewed in [6]), we understand relatively little about the molecular mechanisms that impart risk for sporadic forms of tauopathy—whether primary or secondary. However, because it remains challenging to confidently diagnose sporadic tauopathy in vivo, familial tauopathy (identified via the presence of pathogenic *MAPT* variants in individuals with a family history of neurodegenerative disease) represents a powerful lens through which we can not only elucidate the pathophysiologic processes underlying hereditary tauopathy but also uncover shared processes that may contribute to sporadic tauopathy.

The last decade has witnessed a major revival in interest in immune mechanisms that may modulate risk for neurodegeneration, with a primary focus on microglia, the parenchymal macrophages of the brain (reviewed in [7]). Significantly less progress has been made in elucidating peripheral blood or cerebrospinal fluid (CSF) leukocyte perturbations in—and responses to—neurodegeneration, although this is beginning to change (reviewed in [8–10]). For example, we now know of changes to CD8^+^ T cells in AD and CD4^+^ T cells in Lewy body dementia [11, 12]. In addition, altered phospholipase C-ɣ2 signaling in peripheral lymphocytes has been reported in AD [13]. Beyond lymphocytes, changes in peripheral monocytes (particularly nonclassical [NC] monocytes) have been observed in Parkinson’s disease (PD) [14] and amyotrophic lateral sclerosis (ALS; [15, 16]). In addition, patients with the hereditary white-matter disorder, adult-onset leukoencephalopathy with axonal spheroids, and pigmented glia (ALSP) show striking reductions in peripheral NC monocytes [17]. Given that ALSP is thought to be driven by primary microglial defects (reviewed in [18]), the reduction in peripheral NC monocytes in this disorder suggests shared biology between these two cell types.

High-quality fluid biomarkers are being developed for AD (reviewed in [19, 20]) and FTD (reviewed in [21]), but those that can distinguish between the major neuropathologic division within FTD (i.e., FTLD-tau vs. FTLD due to TDP-43 pathology [FTLD-TDP]) have been lacking [21] until very recently [22]. We reasoned that an in-depth exploration of peripheral immune dysregulation in tauopathy may reveal novel, blood-based biomarkers associated with FTLD-tau. Therefore, in an effort to define the peripheral immune signatures of tauopathy, we carried out single-cell RNA sequencing (scRNA-seq) of peripheral blood mononuclear cells (PBMCs) in individuals with pathogenic *MAPT* variants—who have (or will develop) FTLD-tau pathology—and cognitively normal, non-carrier controls. We identified striking changes in NC monocytes—both in terms of cellular abundance and gene expression—as well as natural killer (NK) cells and other cell types. Moreover, we identified *CX3CR1* as a potentially novel peripheral marker of tauopathy, suggesting parallel changes in *CX3CR1* in microglia and peripheral leukocytes in the course of tau-mediated neurodegeneration. We also identified additional candidate genes whose expression may be altered in the periphery in tauopathy (e.g., *FCGR3A* and *TMEM176A*/*B*). Considering recent findings in ALS and PD, and given that NC monocytes can be detected in the human brain [23], our results add to the weight of evidence suggesting the importance of NC monocytes across a spectrum of neurodegenerative diseases. Taken together, our results indicate that PBMCs represent an accessible and informative tissue source not only for biomarker discovery but also for elucidation of peripheral immune responses in the context of tauopathy.

## Methods

### Study participants

All participants or their surrogates provided written informed consent prior to study participation; all aspects of the studies described here were approved by the University of California, San Francisco (UCSF) institutional review board. Sixteen individuals (*n* = 8 *MAPT* pathogenic variant carriers and *n* = 8 cognitively normal, non-carrier controls) participated in this study. Individuals were recruited as part of ongoing studies of FTD and related neurodegenerative diseases at the UCSF Memory and Aging Center (MAC). All participants underwent a multistep screening with an in-person visit at the MAC that included a neurologic exam, detailed cognitive assessment, medical history, and family history for neurodegenerative disease. Each participant’s study partner was interviewed regarding functional abilities. A consensus team of clinicians then reviewed all participants. In addition, all participants were screened for pathogenic variants in established FTLD genes, including *MAPT*. *MAPT* pathogenic variant carriers had clinical syndromes of bvFTD (*n* = 4), frontal AD (*n* = 1), subjective cognitive impairment (*n* = 1), or were clinically normal and presymptomatic (*n* = 2). Pathogenic *MAPT* variants represented within this study were p.P301L (*n* = 1), p.S305I (*n* = 1), p.S305S (*n* = 1), p.R406W (*n* = 3), and IVS10 + 16 (*n* = 2). Pathogenic variant carriers and cognitively normal controls were sex-matched, and equal numbers of female and male participants were included in the cohort. Age was not significantly different between carrier and control groups, as assessed using an unpaired *t*-test. Demographic information for study participants is included in Table 1.

**Table 1:** Demographic characteristics of cohort

	Control	*MAPT* variant carrier
*n*	8	8
Age, mean (SD)	52.8 (10.0)	54.4 (11.4)
Sex, *n* female	4	4
Clinical syndrome (*n*)	Clinically normal (8)	bvFTD (4), frontal AD (1), subjective cognitive impairment (1), presymp. (2)
*MAPT* variants (*n*)	N/A	p.P301L (1), p.S305I (1), p.S305S (1), p.R406W (3), IVS10 + 16 (2)
Splicing variants, *n*	N/A	4
CDR-SB, mean (SD)	0.0 (0.0)	5.8 (5.7) 7.8 (5.3)^#^

*Abbreviations*: *AD* Alzheimer’s disease, *bvFTD* behavioral variant frontotemporal dementia, *CDR-SB* Clinical Dementia Rating scale Sum of Boxes, *presymp*. presymptomatic, *SD* standard deviation

^#^symptomatic carriers only

### Clinical assessment

Study participants underwent a multistep screening process prior to an in-person clinical evaluation at the UCSF MAC, which included a neurological exam, cognitive assessment, and medical history [24]. Each participant’s study partner was interviewed to assess the participant’s functional abilities. A multidisciplinary team composed of a behavioral neurologist, a neuropsychologist, and a registered nurse established clinical diagnoses for cases according to consensus criteria for FTD and its subtypes [25] or frontal AD [26]. Controls and presymptomatic *MAPT* carriers had a normal neurological exam and a Clinical Dementia Rating scale Sum of Boxes (CDR-SB) [27] score of 0. All controls screened negative for disease-causing pathogenic variation in established AD and FTD genes.

### Cell isolation

Human PBMCs were obtained from study participants at the UCSF MAC. Blood specimens were collected in yellow-top acid-citrate-dextrose vacutainer tubes (BD Biosciences) and processed within 5 h of collection. PBMCs were isolated via Ficoll density gradient centrifugation using Lymphosep separation medium (MP Biomedicals), washed with Ca^2+^- and Mg^2+^-free PBS (Thermo Fisher), and treated with one application of red blood cell lysis buffer (Biolegend). After a final wash step in PBS, PBMCs were diluted to a density of 1.5 × 10^6^ cells/ml in freezing media composed of 10% DMSO in FBS and immediately frozen at − 80 °C. After 2 weeks, samples were transferred to liquid nitrogen for long-term storage. All PBMC samples used in our primary analyses were cryopreserved. Sensitivity analysis involving freshly isolated PBMCs was performed to compare gene expression in paired fresh vs. frozen and thawed samples.

### Single-cell RNA-seq

PBMCs were thawed and prepared for scRNA-seq using the Chromium Single Cell 3’ v2 kit according to the manufacturer’s instructions (10 × Genomics). Samples were processed in two separate batches of eight samples each, with four *MAPT* variant carriers and four controls included in each batch. To further minimize the potential for batch effects, each batch contained equal numbers of samples from female and male participants. After sample thawing, counting, and dilution, PBMCs underwent standard 10 × processing, 3′ gene expression library construction steps, and next-generation sequencing at the UCSF Genomics CoLab and Institute for Human Genetics (IHG).

### Sequencing data processing

For each of the two batches, single-cell 3′ libraries generated from eight samples were pooled and sequenced on one lane of a NovaSeq S4 flow cell. Raw sequencing reads were aligned to GRCh38, and feature-barcode matrices were generated using Cell Ranger version 3.0.2.

### Quality control and clustering

We obtained a total of 7.3 × 10^9^ reads and detected ~ 214,000 cells across the two independent 10 × and sequencing batches, yielding a moderate sequencing depth [28, 29] of ~ 34,000 mean reads/cell. We detected ~ 3700 median UMI counts/cell and ~ 1100 median genes/cell (Additional file 1: Table S1). There were no significant differences in the number of cells captured per sample, the number of reads per sample, or the mean read depth per sample when comparing the *MAPT* pathogenic variant carrier group to the non-carrier control group. Subsequent quality-control (QC) and downstream analysis steps were performed using Seurat v4.1 [30, 31]. QC filtering was applied to individual-sample feature-barcode matrices and consisted of the following steps: (i) genes detected in < 10 cells were removed; (ii) cells with ≤ 500 detected genes were removed; (iii) cells with ≤ 500 counts and those with ≥ 20,000 counts were removed; (iv) cells with mitochondrial mapping percentages ≥ 10 were removed; (v) doublets were identified and removed using DoubletFinder v2.0.3 [32, 33] using the recommended parameter settings. After stringent QC filtering, ~ 181,000 cells remained for downstream analysis (Additional file 2: Table S2).

After QC, we performed the following additional processing steps: (i) we applied sctransform [34]—a method for scRNA-seq count normalization and variance stabilization—at the individual-sample level, including mitochondrial mapping percentage as a covariate [34, 35], to minimize variability due to differences in sequencing depth between samples; (ii) the 16 individual samples were integrated with FindIntegrationAnchors and IntegrateData, specifying “sctransform” as the normalization method and reciprocal principal component analysis (PCA) as the reduction. Subsequently, PCA was performed followed by uniform manifold approximation and projection (UMAP) reduction using the first 30 PCs; clustering was performed using a resolution parameter of 0.5. This resulted in the generation of 21 distinct clusters that were annotated on the basis of marker gene expression, identified using FindMarkers and literature searches. 

### Differential expression analysis

Differential expression analysis was performed using limma [36–39] on individual clusters and sctransform-normalized data, with *MAPT* pathogenic variant carrier status as the contrast and age, sex, and scRNA-seq batch as covariates. To account for multiple testing, a false discovery rate-corrected *p*-value (*p*_FDR_) < 0.05 was considered statistically significant. For visualization of selected differentially expressed genes (DEGs), we used violin plots displaying normalized count data generated via Seurat’s NormalizeData function.

### Cluster proportionality

Cluster proportions were determined for individual samples by dividing the number of cells in a given cluster by the total number of cells in all clusters (after QC filtering) for each individual. Differences in cluster proportionality were assessed visually for all clusters according to *MAPT* variant carrier status. Only the NC monocyte cluster (cluster 11) showed a clear difference in proportionality between carriers and controls. Significance for cluster 11 proportionality, as well as NC monocyte subcluster ratios, was determined by linear modeling, covarying for age, sex, and scRNA-seq batch.

### STRING network analysis

DEGs with *p*_FDR_ < 0.05 and log_2_ fold-changes (LFC) > 0.1 for selected cell clusters were submitted for analysis via the STRING database (v11.5) [40] using the following parameters: the full STRING network was queried; network edge thickness indicated the confidence of the interaction; active interaction sources included databases, experiments, and text mining; a minimum interaction score of 0.4 was required; only query genes/proteins were displayed; disconnected nodes (i.e., DEGs with no interaction partners) were not displayed; gene modules were color-coded according to the results of Markov cluster algorithm (MCL) clustering [41], with modules color-coded within each panel according to their respective gene count. For clarity, within the “Results” section, we refer to specific MCL clusters (containing DEGs) as gene/protein “modules,” while reserving the term “cluster” to refer to cell clusters generated via scRNA-seq analysis.

### Analysis of mouse RNA-seq data

Publicly available mouse RNA-seq data were downloaded from GEO (accession GSE93180) and originally published in [42]. Briefly, hippocampal microglia (Cd11b^+^ myeloid cells) were sorted from 6-month-old male hMAPT-P301S transgenic mice (*n* = 6) or non-transgenic littermates (*n* = 6) using BD FACSAria sorters, then RNA was extracted. RNA-seq libraries were prepared using the Ovation RNA-Seq System V2 (NuGEN). Reads were aligned to the GRCm38 genome using GSNAP and gene counts were acquired using the HTSeqGenie Bioconductor package. Average sequencing depth was 30 million total reads with 8% of reads aligning to exons. We downloaded the available counts data, reanalyzed it using DESeq2 [43], and plotted normalized counts for mouse *Cx3cr1* using ggplot2 [44].

### RNA extraction

To minimize biological variability and facilitate orthogonal validation, for droplet digital (dd)PCR experiments, we used PBMCs isolated from 15 of the 16 participants originally selected for scRNA-seq analysis. One additional cognitively normal control sample was used for ddPCR due to unavailability of one control sample used for scRNA-seq. RNA was extracted from the PBMCs using the RNeasy Micro Kit (Qiagen) and isolated RNA was quantified and its quality was assessed using the RNA 6000 Pico Bioanalyzer kit (Agilent). PBMC RNA samples had RNA integrity number (RIN) values ranging from 9.2 to 9.9, indicating high-quality RNA (Additional file 3: Table S3; [45]).

### Droplet digital PCR

One nanogram of total RNA was used for single-tube reverse transcription (RT) and ddPCR using the One-Step RT-ddPCR Advanced kit (Bio-Rad). Droplets were generated and subsequently analyzed using the QX100 system (Bio-Rad) at the UCSF Center for Advanced Technology (CAT). Reactions were prepared and run essentially according to the manufacturer’s instructions. For steps in which a temperature range was specified, we used the following parameters: RT was performed at 50 °C, annealing/extension occurred at 55 °C, and samples were held at 12 °C in the C1000 thermocycler (Bio-Rad) prior to analysis on the droplet reader. To confirm specificity, we ran the following control reactions: wells lacking RNA but containing all other components and wells lacking reverse transcriptase but containing all other components. PrimePCR ddPCR Gene Expression primer–probe mixes coupled to FAM or HEX (Bio-Rad) were used to amplify specific genes.

### Flow cytometry

Multicolor flow cytometry was performed on thawed PBMC samples using an LSRFortessa (BD). Samples were stained using the following fluorochrome-conjugated antibodies: PE-CF594 mouse anti-human CD14 (BD), APC mouse anti-human CD16 (BD), BV 421 mouse anti-human CD56 (BioLegend), and PE rat anti-human CX3CR1 (BD). Viability was assessed using the LIVE/DEAD Aqua dye (Thermo Fisher). Cells were labeled at room temperature, covered from light for 30 min in a buffer consisting of 1% fetal bovine serum in MACS buffer containing PBS and EDTA (pH 7.2; Miltenyi). After staining, samples were washed three times in the above buffer, then kept on ice until analysis on the LSRFortessa. Compensation was performed using UltraComp eBeads (Thermo Fisher) for the antibodies and ArC amine-reactive compensation beads for LIVE/DEAD Aqua (Thermo Fisher). Control conditions included unstained PBMCs as well as “fluorescence minus one” (FMO) conditions in which a single antibody was omitted. For the primary samples to be analyzed, we acquired data on ~ 100,000 cells falling within the initially defined PBMC gate. All post-acquisition gating and analysis was performed in FlowJo v10 (BD). We used the following sequential gating scheme: (i) debris was excluded via an initial PBMC gate; (ii) live PBMCs were gated by low LIVE/DEAD Aqua fluorescence; (iii) singlets falling along the forward scatter (FSC)-height (-H) vs. FSC-area (-A) diagonal were gated next; (iv) finally, monocytes were gated based on their high side scatter (SSC-A) and CD14 positivity. Monocyte subtypes were gated from a starting population of all monocytes based on their characteristic CD14 vs. CD16 staining patterns. For quantifications, frequencies expressed as a percentage of all PBMCs used the number of cells in the live, singlet PBMC population as the denominator, while frequencies expressed as a percentage of all monocytes used the number of cells in the live, singlet monocyte population as the denominator.

### Analysis of publicly available human brain scRNA-seq dataset

Publicly available human brain scRNA-seq data were downloaded from GEO (accession GSE137444) and originally published in [46]. Briefly, human microglia from patients (*n* = 7) who underwent amygdalohippocampectomy were isolated from the temporal cortex, FACS-sorted, processed for scRNA-seq using the Chromium Single Cell v2 kit (10 × Genomics), and sequenced on a HiSeq 4000 (Illumina). The downloaded counts data were processed and analyzed as described above for our PBMC scRNA-seq data. We used the marker genes identified in [23] to identify the cluster representing human brain CD16^+^ NC monocytes. After identification of the NC monocytes, we assessed cluster-specific expression of *CX3CR1*, *FCGR3A*, *TMEM176A*/*B*, and *C3AR1*.

### Additional statistical analyses

For the analysis of ddPCR data, we performed linear modeling to assess whether *MAPT* carrier status predicted differences in gene expression while covarying for age and sex. Log_2_-transformed absolute concentration data for *CX3CR1*, *FCGR3A*, *TMEM176A/B*, and *C3AR1* (or the ratios of these values with those of reference gene *EEF2*) were used for analyses assuming data normality, while non-transformed data are displayed in the plots. *EEF2* levels can be leveraged as a normalization factor to reduce technical variation across samples. In our assays, we found that the results were unaffected by normalization. To test for differences in mitochondrial genome mapping percentages and differences in the proportions of monocyte subtypes calculated from flow cytometry data, we used Welch’s *t*-test. Differential gene expression for the mouse bulk RNA-seq data was determined using the Wald test implemented in DESeq2. Differences were considered significant at *p* ≤ 0.05 (ddPCR, mitochondrial mapping data, and flow cytometry) or *p*_FDR_ < 0.05 (bulk RNA-seq data). Analyses were performed in R and plots were generated with ggplot2.

## Results

### Reduced nonclassical monocytes in MAPT pathogenic variant carriers

**Fig. 1:**
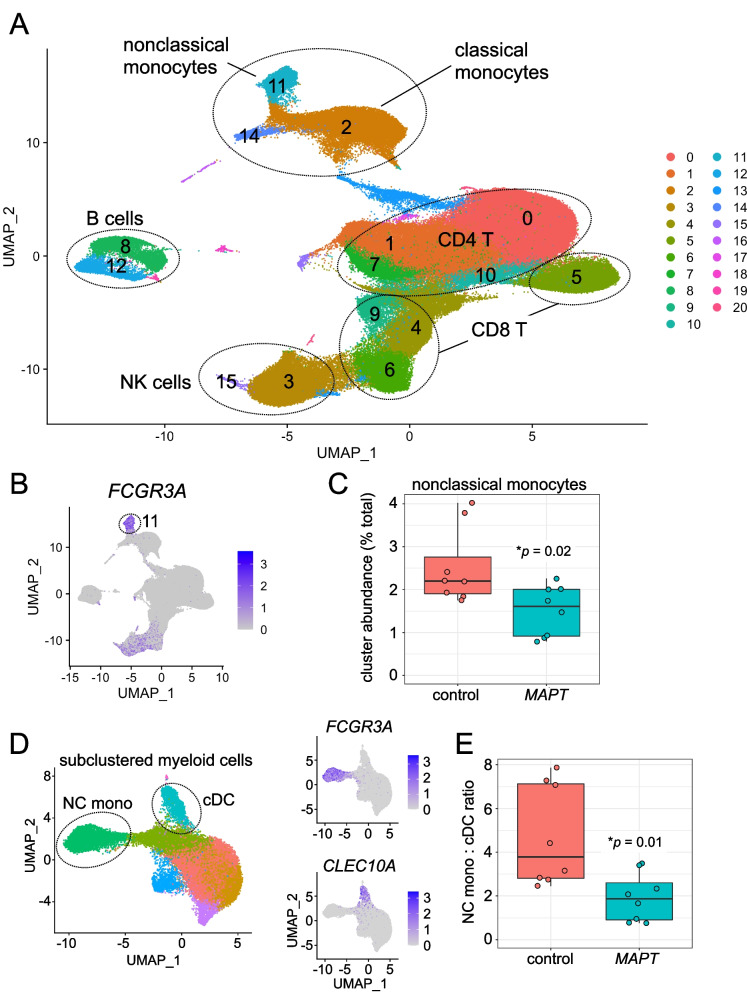
Single-cell RNA-seq reveals reductions in nonclassical monocytes in *MAPT* pathogenic variant carriers. **A** Two-dimensional UMAP plot of ~ 181,000 PBMCs from *MAPT* variant carriers and non-carrier controls, colored by cluster identity. Major cell types are labeled within the plot. **B**, **C** Cluster 11, marked by high *FCGR3A* expression and identified as NC monocytes, was significantly reduced in *MAPT* carriers (*p* = 0.02; data are expressed as percentage of total PBMCs for each sample). **D** Myeloid cells (clusters 2, 11, 14) were subset and re-clustered. NC monocyte and cDC2 subclusters were identified by *FCGR3A* and *CLEC10A* expression, respectively (**D**, right). **E** The ratio of NC monocytes to cDC2 was significantly reduced in *MAPT* variant carriers (*p* = 0.01)

After QC filtering, clustering of ~ 181,000 PBMCs generated 21 primary clusters representing the major lymphoid and myeloid cell types, including CD4^+^ and CD8^+^ T cells, B cells, NK cells, monocytes, and dendritic cells (Fig. 1A; Additional file 4: Fig. S1). As expected, and in contrast to many other FTD-associated genes, *MAPT* expression was barely detectable in PBMCs (Additional file 4: Fig. S2). We reasoned, therefore, that any changes detected in PBMC cell-type proportionality or gene expression in *MAPT* pathogenic variant carriers would most likely represent a response to tau neuropathology or neurodegeneration rather than cell-intrinsic changes occurring directly downstream of variant *MAPT*. Of all PBMC clusters, only one showed a clear, consistent change in abundance in *MAPT* pathogenic variant carriers relative to controls. This cluster (11), which represents *FCGR3A*^+^ (CD16^+^) NC monocytes (Fig. 1A, B), localized in UMAP space near the more-abundant *CD14*^+^ classical monocyte cluster (2) and the *CLEC10A*^+^ conventional dendritic cell (cDC) cluster (14). In particular, NC monocytes, expressed as a percentage of total PBMCs for each participant, were significantly reduced in *MAPT* carriers (Fig. 1C). To gain more fine-grained insight into the nature of this change in abundance, we subsetted and re-clustered myeloid clusters 2, 11, and 14. Assessing the myeloid subclusters (Fig. 1D, left), we discovered that normalizing the NC monocyte subcluster to the *CLEC10A*^+^, *CD1C*^+^ cDC subcluster (corresponding to the most abundant blood cDC population, known as cDC2 [47]; Fig. 1D, lower right panel) yielded better separation between *MAPT* variant carriers and non-carrier controls (Fig. 1E). Further assessing the myeloid subclusters, we found that normalizing NC monocyte numbers to total cDCs (i.e., cDC1 + cDC2) gave a similar result (Additional file 4: Fig. S3A). On the other hand, expressing NC monocyte abundance as a fraction of all monocytes or all myeloid cells did not significantly differentiate carriers from controls (Additional file 4: Fig. S3B, C).

### Differential expression analysis

**Fig. 2:**
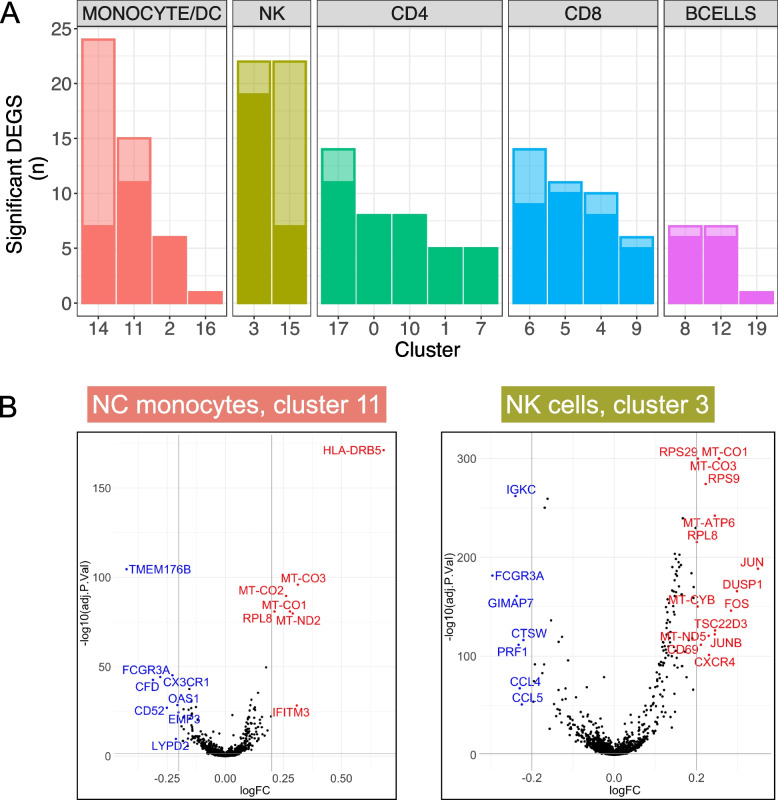
Differential expression in *MAPT* variant carriers by cell cluster. **A** Clusters are grouped by cell type and ranked by the number of DEGs with *p*_*FDR*_ < 0.05 and absolute LFC > 0.2. Differential expression was determined in *MAPT* variant carriers relative to non-carrier controls while covarying for age, sex, and scRNA-seq batch. Solid-colored portions of the bars indicate DEGs shared by at least one other cluster, while the translucent portions indicate DEGs unique to a given cluster. cDCs (cluster 14), NK cells (clusters 3 and 15), and NC monocytes (cluster 11) had the highest numbers of DEGs with absolute LFC > 0.2. **B** Volcano plots of the NC monocyte cluster and major NK cell cluster; DEGs with absolute LFC > 0.2 are labeled in blue (downregulated) or red (upregulated). Several NK cell DEGs (right) with − log_10_(*p*_FDR_) values > 300 were set to 300 for visualization purposes

We next performed differential expression analysis on each of the primary cell clusters, comparing *MAPT* variant carriers to non-carrier controls and adjusting for age, sex, and scRNA-seq batch. Focusing initially on DEGs with absolute LFC > 0.2, we determined that cDC, NC monocyte, and NK cell clusters had the highest number of DEGs (Fig. 2A, B). We further characterized the DEGs of NC monocytes and NK cells by querying the STRING database [40] for functional and physical interactions, now using a more-permissive LFC cutoff of 0.1 to facilitate population of the gene interaction network (see Additional file 5: Table S4 for DEG lists for all clusters).

### STRING network analysis

**Fig. 3:**
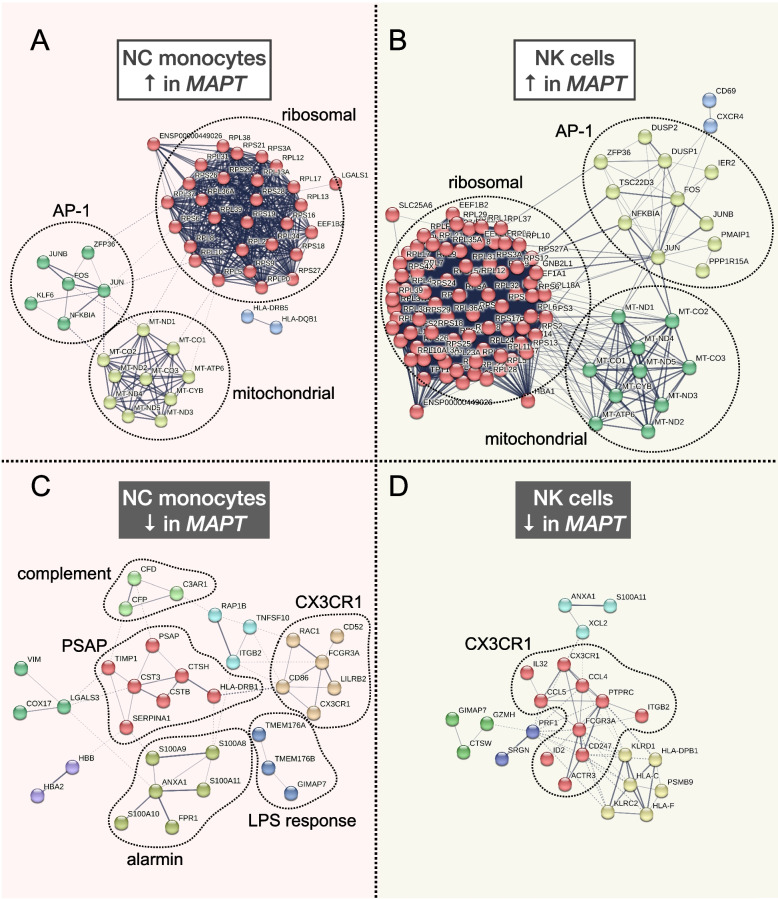
STRING interaction networks reveal relationships among nonclassical monocyte and natural killer cell differentially expressed genes.** A**, **B** Upregulated DEGs in NC monocytes and NK cells had similar overall network architecture, with large ribosomal and mitochondrial modules, and a third module containing members of the AP-1 transcription factor, among other genes. **C** The downregulated DEGs in NC monocytes contained a module featuring *CX3CR1* and *FCGR3A* as members, in addition to modules harboring genes involved in LPS response, the alternative and classical complement cascades, the *S100* alarmin molecules, as well as *PSAP*. **D** Downregulated DEGs in NK cells also featured a large module featuring both *CX3CR1* and *FCGR3A*. All DEGs with *p*_*FDR*_ < 0.05 and absolute LFC > 0.1 from clusters 3 and 11 were input into the STRING database as described in the “Methods” section. Modules are colored according to the results of MCL clustering

*MAPT* carriers showed striking upregulation of many ribosomal and mitochondrial genes that, respectively, formed large interaction modules (Fig. 3A, B). The biological significance of these coordinately upregulated DEGs is unclear, but tau is known to interact with and affect multiple ribosomal subunits [48–51] and mitochondrial proteins [51, 52]. Because the identified mitochondrial DEGs represent a subset of the mitochondrial genes used during QC (see “Methods”) to filter out putatively damaged cells [35], we considered the possibility that—despite the removal of cells with high mitochondrial mapping percentages (≥ 10%)—the apparent upregulation of mitochondrial DEGs may nevertheless be associated with higher mitochondrial mapping percentage in *MAPT* variant carriers. Consistent with this possibility, mitochondrial mapping percentage was subtly but significantly higher in *MAPT* carriers in the NC monocyte and NK cell clusters (11 and 3, respectively; Additional file 4: Fig. S4A, B). On the other hand, mitochondrial DEGs were absent from the cDC cluster (14; Additional file 4: Fig. S4D; Additional file 5: Table S4)—despite this cluster having the highest overall number of DEGs with LFC > 0.2 (Fig. 2A)—and mitochondrial mapping percentage was not significantly increased in variant carriers in this cluster (Additional file 4: Fig. S4C). This suggests (i) cell-type-specific and, presumably, biologically relevant dysregulation of mitochondrial genes in *MAPT* variant carriers, consistent with [51]; and (ii) that the upregulation of mitochondrial DEGs in the NC monocyte and NK cell clusters is associated with increased mitochondrial mapping percentage. To test the latter possibility, we next included mitochondrial mapping percentage as an additional covariate in the differential expression analyses, and, as expected, nearly all mitochondrial DEGs that previously had estimated LFCs > 0.1 no longer passed this threshold (not shown). Thus, the presence of mitochondrial DEGs is associated with increased mitochondrial mapping percentage. Importantly, coordinated upregulation of mitochondrial genes could lead to subtle shifts in mitochondrial mapping percentage, and therefore the causality underlying this relationship is unclear.

The other major upregulated STRING module found in both NC monocytes and NK cells contained members of the AP-1 transcription factor family [53], including *FOS*, *JUN*, and *JUNB* (Fig. 3A, B). Multiple members of this module (*FOS*, *DUSP1*) were previously found to be upregulated via bulk measurements of both PBMCs and hippocampus in AD [54], suggesting consistent dysregulation of AP-1 transcription factor genes in both primary and secondary tauopathies. *NFKBIA*, encoding the NF-κB inhibitor-ɑ, also appears in this module in both NC monocytes and NK cells, and this gene is highly upregulated by treatment of microglia with tau paired-helical filaments [55] and fibrils [56] and in the brain in late-onset AD [57].

**Fig. 4:**
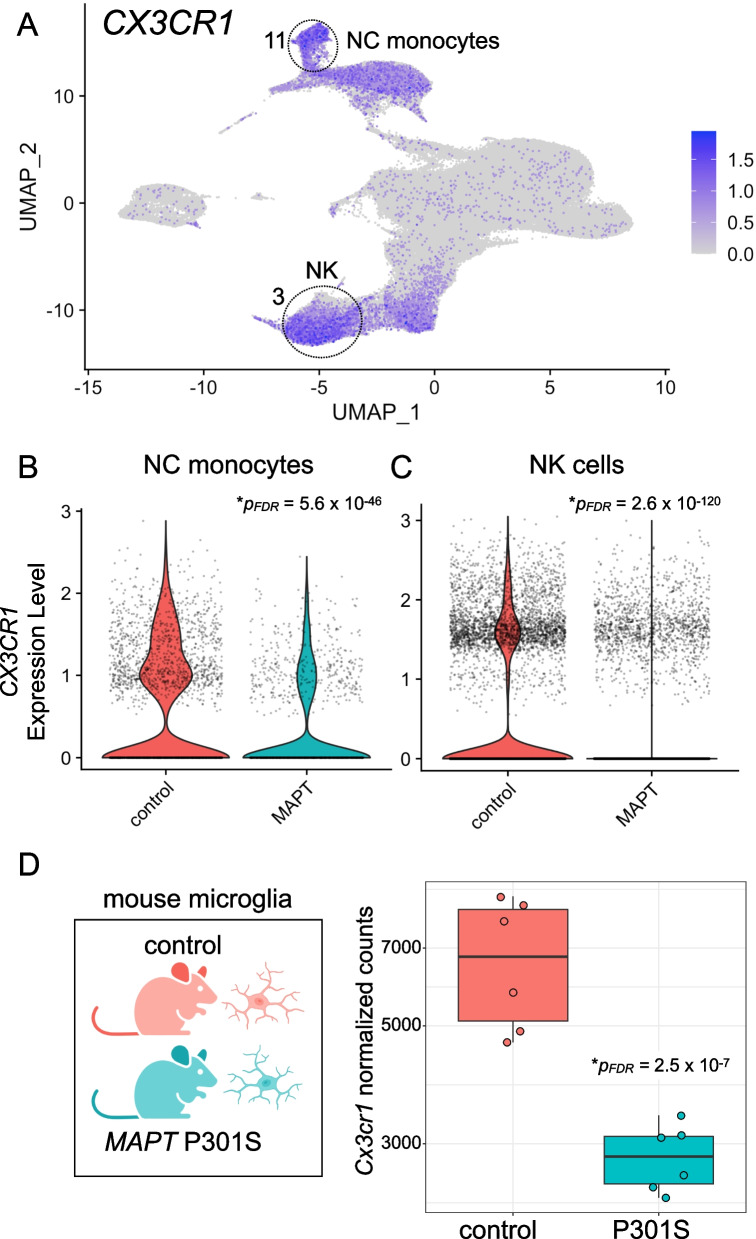
*CX3CR1* expression is reduced in peripheral myeloid and lymphoid cells in familial tauopathy. **A** *CX3CR1* is robustly expressed in both NC monocytes (cluster 11) and NK cells (cluster 3). NC monocytes (**B**; *p*_*FDR*_ = 5.6 × 10^−46^) and NK cells (**C**; *p*_*FDR*_ = 2.6 × 10^−120^) both show significantly reduced expression of *CX3CR1* in *MAPT* pathogenic variant carriers. **D** Reanalysis of publicly available bulk RNA-seq data from mouse hippocampal CD11b^+^ microglia demonstrated a significant reduction (*p*_FDR_ = 2.5 × 10^−7^) of *Cx3cr1* in the *MAPT* P301S model

In terms of significantly downregulated genes, both NC monocytes and NK cells contained a module populated by *FCGR3A* and *CX3CR1* (Fig. 3C, D). Although *FCGR3A* is an established marker of NC monocytes, it is also expressed by a subset of NK cells (Fig. 1B). *CX3CR1* is highly expressed by both NC monocytes and NK cells (Fig. 4A) and, strikingly, is a well-known modulator of tau pathophysiology in the brain [58–60]. Additional downregulated modules in NC monocytes included those containing components of the complement pathway (*CFD*, *CFP*, *C3AR1* [61, 62]), members of the S100 alarmin family (*S100A8*-*11* [63, 64]), highly lipopolysaccharide (LPS)-responsive microglial genes (*TMEM176A*, *TMEM176B* [65]), and the lysosomal gene *PSAP*, which promotes pro-inflammatory activity in monocytes [66] (Fig. 3C). Collectively, the downregulation of these gene modules in *MAPT* pathogenic variant carriers suggests possible dampening of latent pro-inflammatory pathways in NC monocytes. Of note, this apparent phenotypic change also occurred within a diminished population of circulating NC monocytes.

### Validation of select differentially expressed genes

We next sought to validate a handful of DEGs which showed robust changes by differential expression analysis, often in multiple cell types, focusing on those deemed most likely to be biologically relevant to tau pathophysiology. In particular, we selected the following genes for validation via an orthogonal technique, ddPCR: *CX3CR1*, *FCGR3A*, *TMEM176A*, *TMEM176B*, and *C3AR1*. As noted above, *CX3CR1* has many well-established connections to tau pathology via its role in microglia. However, to our knowledge, its role in peripheral myeloid cells has not been studied in the context of neurodegenerative disease. *FCGR3A* not only serves as a marker gene for the significantly reduced population of NC monocytes, but was also significantly downregulated in both NC monocytes and NK cells of *MAPT* carriers. *TMEM176A*/*B* are less well known but have been shown to be extremely responsive to LPS treatment in human microglia [65], are homologs of the *MS4A* gene family involved in risk for AD [67], and have also been shown to be dysregulated in PBMCs from AD patients [54]. Finally, *C3AR1* has been implicated as a key player in the spread of tau neuropathology in mouse models [68], while the complement system more generally is thought to be a key regulator of neuronal loss in primary tauopathy as well as AD [69]. We first discuss validation of the *CX3CR1* finding below.

### Reduced CX3CR1 expression in familial tauopathy

**Fig. 5:**
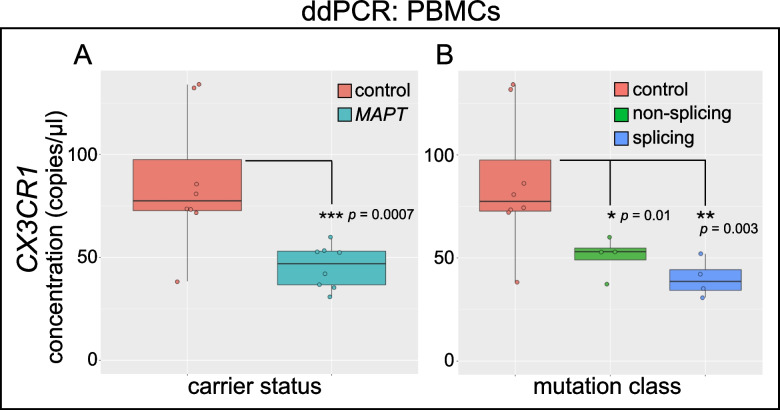
Confirmation of reduced *CX3CR1* expression in *MAPT* variant carrier PBMCs via ddPCR. RNA was isolated from PBMCs from *MAPT* variant carriers and healthy, non-carrier controls; gene expression was determined by RT-ddPCR. **A** *CX3CR1* was significantly reduced in *MAPT* carrier PBMCs (*p* = 0.0007) relative to controls. **B** Separation of samples according to *MAPT* variant class (non-splicing and splicing) reveals that *CX3CR1* was significantly reduced in both groups, relative to controls (non-splicing, *p* = 0.01; splicing, *p* = 0.003)

*CX3CR1* showed robust expression in NC monocytes and NK cells (Fig. 4A), and its expression was significantly reduced in both cell types in *MAPT* pathogenic variant carriers (Fig. 4B, C). To determine whether downregulation in peripheral leukocytes was mirrored by changes in brain myeloid cells in the context of tauopathy, we analyzed a publicly available brain RNA-seq dataset [42] derived from *MAPT* P301S mouse hippocampal microglia. P301S microglia also displayed downregulation of *Cx3cr1* (Fig. 4D), indicating consistent *CX3CR1* dysregulation between mouse microglia and human peripheral immune cells in the context of tauopathy. Importantly, the P301S mutation used in this mouse model was not represented in our cohort, suggesting that the reduced peripheral *CX3CR1* observed here may be a general feature of familial tauopathy and may therefore occur downstream of pathogenic *MAPT* variants beyond those directly assessed in this work. To validate the change in *CX3CR1* expression in peripheral immune cells, we isolated PBMC RNA from *MAPT* pathogenic variant carriers and non-carrier controls and performed ddPCR. ddPCR confirmed the reduction in *CX3CR1* (Fig. 5A). Normalizing the *CX3CR1* signal to reference gene *EEF2* gave similar results (Additional file 4: Fig. S5A, B). When *MAPT* variant carriers were stratified by variant class (splicing vs. non-splicing), both groups showed significant reductions in *CX3CR1* (Fig. 5B), suggesting that multiple mechanistic forms of familial tauopathy converge on perturbation of *CX3CR1* expression.

### Diminished FCGR3A expression in MAPT pathogenic variant carriers

**Fig. 6:**
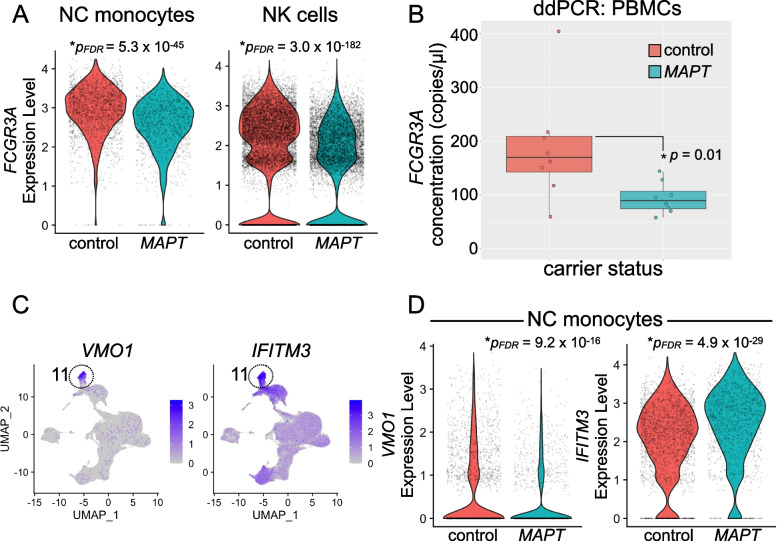
Analysis of nonclassical monocyte marker genes in *MAPT* variant carriers. **A** *FCGR3A*, the NC monocyte marker gene encoding CD16, is robustly expressed not only in NC monocytes but also in NK cells. *FCGR3A* is significantly reduced in both NC monocytes (left; *p*_*FDR*_ = 5.3 × 10^−45^) and NK cells (right; *p*_*FDR*_ = 3.0 × 10^−182^) in *MAPT* pathogenic variant carriers. **B** ddPCR confirmed a reduction in *FCGR3A* reduction in *MAPT* variant carrier PBMCs (*p* = 0.01). **C** Additional genes expressed specifically (*VMO1*, left) or enriched in (*IFITM3*, right) NC monocytes showed significant alterations (**D**) in *MAPT* variant carrier NC monocytes. **D** *VMO1* (left) was significantly reduced (*p*_*FDR*_ = 9.2 × 10^−16^), while *IFITM3* (right) was significantly increased (*p*_*FDR*_ = 4.9 × 10^−29^) in *MAPT* carriers

We next focused on validation of *FCGR3A*. We predicted that *FCGR3A* would show a robust decrease in expression in *MAPT* variant carriers in bulk PBMC RNA given that (i) NC monocytes are reduced in abundance in carriers (Fig. 1C), and (ii) *FCGR3A* expression was reduced in both NC monocytes and NK cells by scRNA-seq differential expression analyses (Figs. 6A and 2B). Indeed, ddPCR analysis showed a significant reduction in *FCGR3A* expression in *MAPT* pathogenic variant carrier PBMCs (Fig. 6B). The observed reduction likely reflects both reduced expression of *FCGR3A* and reduced levels of NC monocytes, which express *FCGR3A* at high levels. As with *CX3CR1*, normalization of the *FCGR3A* signal to reference gene *EEF2* did not affect the results (Additional file 4: Fig. S5C). We also analyzed additional marker genes of the NC monocyte cluster, including *VMO1* and *IFITM3*. *VMO1*, which is specifically expressed in cluster 11 (consistent with [17]), and *IFITM3*, which is enriched in cluster 11, both showed significant differential expression in *MAPT* variant carriers (Fig. 6C, D; Additional file 5: Table S4), indicating changes in multiple NC monocyte marker genes. Because the ddPCR and scRNA-seq studies described here depend on the use of cryopreserved PBMCs, we tested whether cryopreservation had a major effect on the measured expression levels of *CX3CR1* and *FCGR3A*. Direct comparison by ddPCR of paired, fresh vs. frozen/thawed samples suggested that cryopreservation was associated with only a modest, ~ 15–20% reduction in the apparent expression of these transcripts (Additional file 4: Fig. S6A, B). Cryopreservation, therefore, was not associated with a major change in the levels of *CX3CR1* and *FCGR3A*.

### Analysis of TMEM176A/B in familial tauopathy

**Fig. 7:**
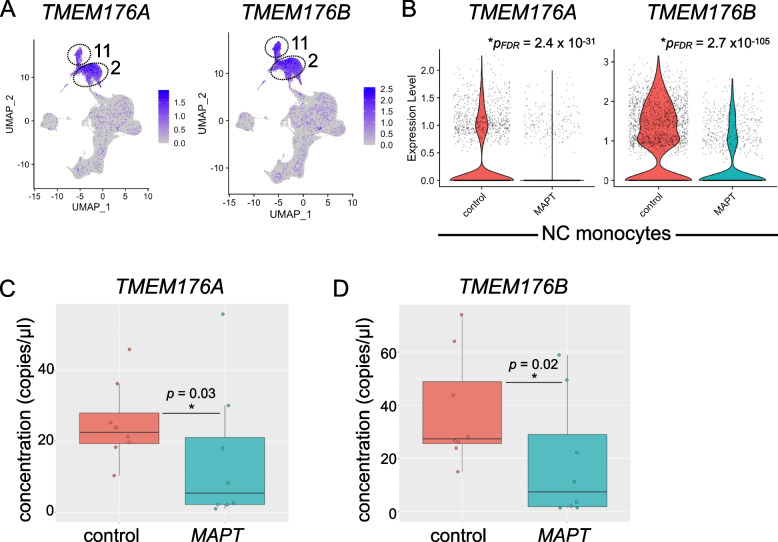
Analysis of *TMEM176A*/*B* in *MAPT* pathogenic variant carriers.** A** *TMEM176A*/*B* are highly expressed in both classical (cluster 2) and NC (cluster 11) monocytes. **B** *TMEM176A*/*B* are significantly reduced in NC monocytes (*TMEM176A*, *p*_*FDR*_ = 2.4 × 10^−31^; *TMEM176B*, *p*_*FDR*_ = 2.7 × 10.^−105^) from *MAPT* carriers. These genes were similarly reduced in *MAPT* carrier classical monocytes (cluster 2; Additional file 5: Table S4). The reduction in *TMEM176A* (**C**) and *TMEM176B* (**D**) in *MAPT* variant carriers was confirmed using bulk PBMC RNA and ddPCR (*TMEM176A*, *p* = 0.03; *TMEM176B*, *p* = 0.02)

*TMEM176A*/*B* represent poorly characterized genes of the extended *MS4A* family [67] thought to be involved in microglial LPS response [65], antigen presentation [70], and inflammasome regulation [71]. *TMEM176A*/*B* were highly expressed in classical and NC monocytes (clusters 2 and 11; Fig. 7A) and were strongly downregulated in *MAPT* variant carrier NC monocytes (Fig. 7B). Reduced expression of *TMEM176A* and *TMEM176B* was confirmed via ddPCR of PBMC RNA (Fig. 7C, D). The levels of *TMEM176A* and *TMEM176B*, as detected by ddPCR, were tightly associated with one another, and a subset of *MAPT* pathogenic variant carriers (5 of 8) displayed lower expression of both genes than any non-carrier controls (Additional file 4: Fig. S5D).

### Analysis of C3AR1 in MAPT pathogenic variant carriers

**Fig. 8:**
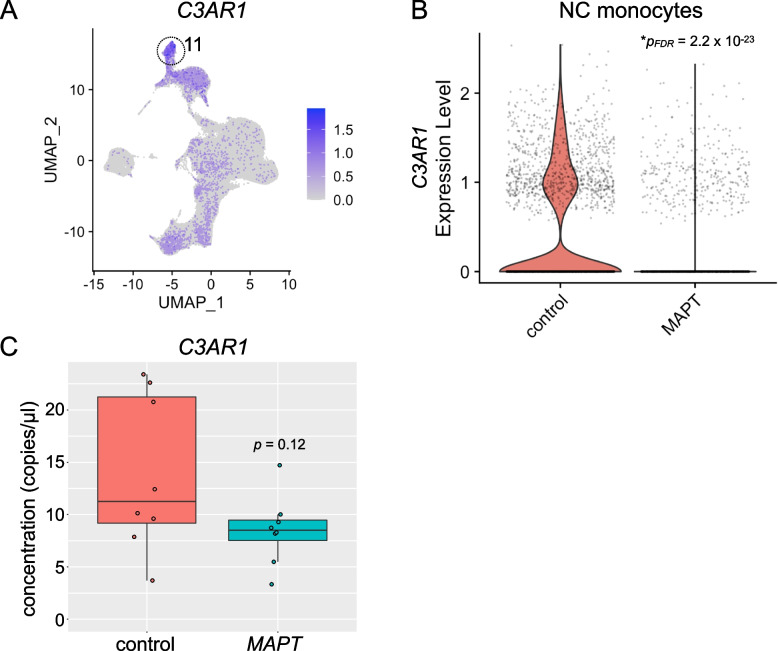
Potential dysregulation of *C3AR1* in *MAPT* pathogenic variant carriers.** A** *C3AR1* expression was enriched in the NC monocyte cluster (11). **B** *C3AR1* expression in NC monocytes was significantly reduced in *MAPT* variant carriers (*p_*FDR*_ = 2.2 × 10.^−23^). **C** ddPCR analysis of PBMC RNA revealed a trend toward reduced expression of *C3AR1* in *MAPT* variant carriers which did not reach significance (*p* = 0.12)

Finally, we examined the expression of *C3AR1* given its importance in models of tauopathy [68]. *C3AR1* expression was enriched in NC monocytes (Fig. 8A) and was strikingly reduced in this cell type in *MAPT* variant carriers (Fig. 8B). *C3AR1* expression, as measured by ddPCR of PBMC RNA, trended toward reduction but did not achieve significance (Fig. 8C). However, given the importance of this gene and the complement pathway more generally in tauopathy and AD, analysis of peripheral *C3AR1* expression in larger, better-powered cohorts is still warranted.

### Validation of the reduction in nonclassical monocytes via flow cytometry

**Fig. 9:**
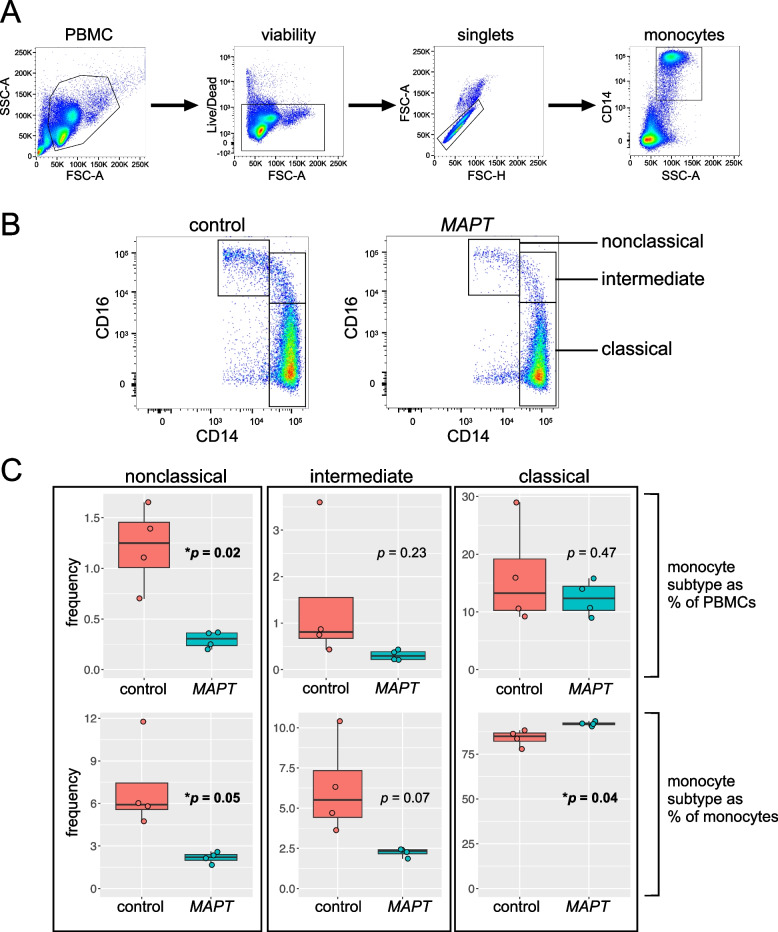
Validation of the reduction in nonclassical monocytes via flow cytometry.** A** Gating scheme for identification and analysis of monocyte subtypes. PBMCs were gated as follows: debris was excluded, non-viable cells were excluded, then doublets were excluded. Next, monocytes were gated based on their high side scatter and CD14 expression. **B** Monocyte subtypes were gated based on their characteristic CD14 and CD16 expression, with classical monocytes having high CD14 expression and low CD16 expression, intermediate monocytes having high CD14 expression and moderate-to-high CD16 expression, and NC monocytes having low CD14 expression and high CD16 expression. **C** Quantification of the frequency of NC (left), intermediate (center), and classical monocytes (right), either as a percentage of PBMCs (top row) or all monocytes (bottom row). NC monocytes were reduced in *MAPT* pathogenic variant carriers as a fraction of PBMCs (upper left, *p* = 0.02) and as a fraction of monocytes (lower left, *p* = 0.05). Intermediate monocytes (center) showed a trend toward reduction relative to both PBMCs and monocytes. Classical monocytes (right) showed no change as a fraction of PBMCs but were significantly increased in *MAPT* pathogenic variant carriers as a fraction of all monocytes

To determine whether a central finding of the scRNA-seq analysis could be directly validated via an independent method, we performed multicolor flow cytometry analysis on PBMCs from *MAPT* pathogenic variant carriers and non-carrier controls. PBMCs, monocytes, and monocyte subtypes were gated as illustrated in Fig. 9A and B and as described in detail in the “Methods” section. Importantly, this analysis enabled us to confirm a significant reduction in NC monocytes (characterized by low CD14 expression and high CD16 expression) as a percentage of all PBMCs (*p* = 0.02; Fig. 9C, upper left). We also observed a marginally significant reduction in NC monocytes expressed as a percentage of all monocytes (*p* = 0.05; Fig. 9C; lower left). Intermediate monocytes (characterized by high CD14 levels and moderate-to-high CD16 levels) showed a trend toward reduced abundance, relative to either all PBMCs or all monocytes, but this difference did not reach significance (Fig. 9C, middle panels). Finally, classical monocytes (characterized by high CD14 levels and low expression of CD16) showed no change as a fraction of all PBMCs, but, as expected—given the relative reductions in NC and intermediate monocytes—showed a significant increase when expressed as a percentage of all monocytes (*p* = 0.04; Fig. 9C, lower right). No differences in viability were detected between *MAPT* pathogenic variant carriers and non-carrier controls (Additional file 4: Fig. S7). An alternative gating scheme that enabled the inclusion of additional NC monocytes with lower levels of CD14 expression resulted in very similar findings (Additional file 4: Fig. S8). Taken together, our observations are consistent with a bona fide decrease in the frequency of NC monocytes in *MAPT* pathogenic variant carriers, and this alteration does not appear to be secondary to an absolute increase in classical monocyte abundance. Finally, we also asked whether alterations in cell-surface expression of CX3CR1 and CD16 could be detected via flow cytometry on NC monocytes or NK cells. To do so, we measured the median fluorescence intensity (MFI) for CX3CR1 and CD16 in each cell population, but we did not observe a difference between *MAPT* carriers and non-carrier controls (data not shown). We infer from this that the ~ 50% reduction in transcript abundance for *CX3CR1* and *FCGR3A* is insufficient to alter the cell-surface levels of their encoded proteins. Alternatively, there may be a more general divergence between the dysregulation of these transcripts and their encoded proteins. Future work will test these two possibilities.

### Analysis of gene expression in human brain nonclassical monocytes

To begin to understand why peripheral NC monocytes are affected in familial tauopathy, we asked whether these cells have access to the human central nervous system (CNS). Indeed, very recent work has suggested an important role for human NC monocytes in the CSF during aging [72], and a recent analysis [23] of publicly available scRNA-seq data [46, 73] has also identified these cells in the human temporal cortex. We downloaded and reanalyzed this dataset (accession GSE137444, [46]) and similarly identified a clear NC monocyte population (cluster 13 in our reanalysis; Additional file 4: Fig. S9A), thus recapitulating the findings from [23]. This cluster was unique in its high expression of NC monocyte marker genes such as *LYZ*, *S100A8*, *S100A9*, and *FCN1* (marker genes from [23]; Additional file 4: Fig. S9B). We next assessed expression levels of the DEGs that we focused on in this work—*CX3CR1*, *FCGR3A*, *TMEM176A*/*B*, and *C3AR1*. We detected robust expression of all genes except *TMEM176A* in the brain NC monocyte cluster (Additional file 4: Fig. S9C). Collectively, these findings suggest that NC monocytes have ready access to the human brain and potentially explain why robust changes in NC monocytes can be observed in the context of tauopathy—that is, they may have direct access to tissue harboring tau pathology. Future functional studies will be needed to determine NC monocytes’ potential to impact the course of tauopathy. In particular, depletion of NC monocytes in mouse models of tauopathy would enable direct testing of the role these cells play in the development of tau neuropathology.

## Discussion

This study represents an effort to discover novel, blood-based biomarkers of tauopathy, and—more broadly—to begin to understand the nature of the peripheral leukocyte response to primary tauopathy. To this end, we conducted an unbiased scRNA-seq survey of ~ 181,000 PBMCs in carriers of pathogenic *MAPT* variants and healthy, non-carrier controls. In doing so, we uncovered novel, peripheral blood transcriptomic signatures of familial tauopathy at single-cell resolution. In particular, we observed a significant reduction in circulating NC monocytes and numerous DEGs that were particularly enriched in specific myeloid and NK cell clusters. Next, we validated changes in several candidate DEGs selected on the basis of plausible biological relevance. These included *CX3CR1*, *FCGR3A*, and *TMEM176A/B*. In addition, *C3AR1*, although not found to be significantly reduced in *MAPT* carriers by ddPCR, still trended toward reduced expression.

The differential expression of *CX3CR1* observed by scRNA-seq was not only replicated via ddPCR analyses but also confirmed using a publicly available mouse microglia bulk RNA-seq dataset derived from the *MAPT* P301S model. This suggests *CX3CR1* may have potential utility as a peripheral biomarker of tauopathy and warrants further study in larger cohorts. Intriguingly, mouse *Cx3cr1* has been known for over a decade to control the levels of NC monocytes [74–76]. In addition, mouse *Cx3cr1*-mediated control of NC monocyte levels has more recently been suggested to modulate the innate immune response to traumatic brain injury [77]. Collectively, these findings suggest that the reduction in *CX3CR1* expression we observe in *MAPT* pathogenic variant carrier NC monocytes (on a per-cell basis) may be directly related to the concomitant reduction in NC monocyte abundance also observed in *MAPT* carriers.

Quite aside from the role of *Cx3cr1* in controlling circulating NC monocyte levels, *Cx3cr1* exerts a well-established, microglia-mediated modulatory effect on tau neuropathology in mouse models [58–60, 78–84]. In particular, deletion of *Cx3cr1* promotes hallmark neuropathological features of tauopathy including tau hyperphosphorylation and aggregation [58–60, 84]. More-recent studies on induced pluripotent stem cell-derived microglia-like cells have confirmed the importance of *CX3CR1* in regulating human microglial homeostasis [85], consistent with *Cx3cr1*’s established role in promoting the homeostatic microglial phenotype in mice [86]. Given that microglial homeostasis is dysregulated in tauopathy [87–90], our novel findings—coupled with the well-defined relationship between *CX3CR1* and tauopathy—provide a promising foundation for further investigation of this gene as a peripheral biomarker of tauopathy.

As noted above, we observed a significant reduction in NC monocytes in *MAPT* carriers relative to non-carrier controls. NC monocytes are recruited to sites of vascular damage, infection, or inflammation to patrol the local environment [76]. At these compromised sites, chemoattractant factors are released, and NC monocytes respond through the expression of cognate receptors, including CX3CR1 [76]. Strikingly, NC monocytes are thought to be reduced in peripheral blood in a variety of neurodegenerative diseases, including ALS [15, 16], PD [14], and the adult-onset, hereditary leukoencephalopathy, ALSP [17]. Conversely, NC monocyte levels may be increased in the CSF in PD, suggesting a possible shift of this monocyte population from blood to CSF in the context of neurodegeneration [91]. Considering these findings, and given that (i) ALS exists along a spectrum with FTD [92]; (ii) a portion of PD risk is mediated by variation near the *MAPT* locus [93, 94]; and (iii) ALSP can manifest clinically as FTD [18], our finding of reduced NC monocytes in familial tauopathy both strengthens and extends the purported relevance of this monocyte population in neurodegenerative disease.

We observed significantly reduced expression of the canonical NC monocyte marker gene *FCGR3A* as well as significant alterations in two additional NC monocyte marker genes (*VMO1* and *IFITM3*) in *MAPT* pathogenic variant carriers by scRNA-seq. Given that *FCGR3A* expression was reduced not only in NC monocytes but also in NK cells and considering that NC monocyte abundance was simultaneously reduced, we reasoned that bulk assessment of PBMC RNA via ddPCR would be well suited to detect reduced expression of *FCGR3A* in *MAPT* carriers, and, indeed, this is what we found. This cellular phenotype suggests several possibilities. First, the reduced abundance of NC monocytes and diminished expression of *FCGR3A* on the remaining NC monocytes could reflect migration of mature NC monocytes (that express the highest levels of *FCGR3A*) out of the blood and into another compartment (e.g., CSF or brain). Second, our findings could reflect impaired survival of NC monocytes in tauopathy. Third, lower *FCGR3A* levels might reflect impaired differentiation of NC monocytes from classical or intermediate monocytes (the latter of which express intermediate levels of *FCGR3A*). We favor the first two possibilities, as the latter scenario would be expected to involve accumulation of other classes of monocytes, which we did not observe.

Our differential expression analyses identified cDCs (cluster 14) as the cell type with the highest number of DEGs with absolute LFC > 0.2. Although we did not focus on cDCs for our validation studies, we did find that normalizing NC monocyte abundance to cDC abundance enabled a clear separation of *MAPT* carriers from non-carrier controls. This finding begets the question: what is the biological link connecting NC monocytes to cDCs? Emerging literature has highlighted several intriguing connections between NC monocytes and particular subsets of DCs. For example, a putative DC subpopulation, identified transcriptomically and originally termed DC4 [95], is now considered to probably represent a subset of NC monocytes rather than DCs [47, 96, 97]. In addition, pathogenic variants in *STAT3* have revealed this gene’s role in regulating the production of both NC monocytes and the less-abundant cDC population, cDC1 [98]. Although we could detect the rare cDC1 population in our dataset, it became apparent only upon myeloid cell re-clustering (myeloid subcluster 7, marked by *CLEC9A* expression), and these cells were too sparse to enable us to accurately gauge their abundance or use for normalization purposes. On the other hand, it remains unclear precisely how NC monocytes are biologically related to the more-abundant cDC2 population. Nevertheless, normalizing NC monocyte abundance to either cDC2 or total cDC abundance enabled a clear separation of *MAPT* variant carriers from healthy controls. Future studies in larger cohorts will be required to determine the precise quantification metric for NC monocytes that best differentiates *MAPT* carriers from controls. It will also be important to establish whether this finding extends to sporadic forms of tauopathy; this seems likely given that similar phenomena have been reported in disparate neurodegenerative diseases.

Recently published work indicates robust transcriptional changes in NC monocytes within the CSF during healthy aging and in the context of cognitive impairment [72]. An additional recent finding [23], which we confirm here (Additional file 4: Fig. S9), indicates that NC monocytes can also be found in the human temporal cortex. Given that the precise role of human NC monocytes in health and disease is still largely unknown [76], it remains unclear what a reduction in peripheral NC monocytes means vis-à-vis tauopathy. If the observed reduction in peripheral NC monocytes is accompanied by a corresponding increase in CNS NC monocytes—and if they contribute to heightened neuroinflammation—they are likely to play a net detrimental role in neurodegeneration. However, much work remains to be done to test these possibilities.

Beyond myeloid cells, our work also highlights a potentially novel role for NK cells in primary tauopathy. In particular, NK cells had a large number of DEGs with LFC > 0.2, and our findings implicating *CX3CR1* expression not only in NC monocytes but also in NK cells as a candidate peripheral biomarker of familial tauopathy is complemented by recent research suggesting an important yet previously unappreciated role for NK cells in a mouse model of AD [99]. In addition, NK cell recruitment to the CNS has been observed in ALS as well as ALS models [100]. Collectively, the available data suggest a detrimental role for NK cells in ALS and AD. More broadly, in the experimental autoimmune encephalomyelitis model of multiple sclerosis, NK cell migration into the CNS is mediated in part by CX3CR1-dependent recruitment [101, 102], suggesting that the differential expression of *CX3CR1* in NK cells that we observed in our study could plausibly affect NK cell recruitment to the CNS in primary tauopathy.

Reliable biomarkers can improve diagnostic acumen and enable elucidation of specific forms of neuropathology underlying clinical dementia syndromes. For example, examination of brain structure and function via neuroimaging is a powerful method for the determination of neurodegenerative disease etiology. The use of positron emission tomography (PET) imaging, in particular, with radiotracers that bind to aggregated forms of tau has facilitated the in vivo detection of tau neuropathology in individuals with AD (reviewed in [19, 103, 104]). However, tau-PET tracers do not bind strongly to most forms of FTLD-tau pathology and may exhibit off-target binding in individuals with FTLD-TDP pathology [103, 104]. Alternatively, the use of CSF- and blood-based protein biomarkers holds great promise for AD [19, 20, 105] and FTD [21, 106], although in the case of FTD, we still cannot discriminate between underlying FTLD-tau and -TDP pathology. Important limitations apply to several of these methods. In particular, PET imaging is costly and available only at specialized medical centers, and CSF collection requires invasive lumbar puncture. In contrast to these methods, peripheral blood biomarkers are easy to collect and, when coupled with analytic techniques such as ddPCR, may eventually enable low-cost alternatives to today’s better-developed biomarkers.

Despite numerous advances described above, our study has several important limitations. First, due to the significant expense of scRNA-seq and our desire to capture a relatively large number of PBMCs per individual, we necessarily used a small cohort for this study. We also opted to confirm gene expression findings via an orthogonal technique using bulk PBMC RNA measurements in essentially the same cohort that was used for the scRNA-seq analysis. While we employed this strategy to minimize the possibility that technical artifacts drove discovery of the candidate genes we characterized, it will be important to evaluate the generalizability of our findings in larger cohorts. Larger cohorts will also enable us to determine whether any of the dysregulated genes identified here are differentially sensitive to pathogenic *MAPT* variants that affect splicing, relative to those that do not, as potentially suggested for *CX3CR1* (Fig. 5B). In addition, future work that measures absolute numbers of NC monocytes in familial tauopathy would be a valuable addition to the field, as the scRNA-seq and flow cytometry data reported here show relative reductions in NC monocyte frequency in *MAPT* pathogenic variant carriers. Another issue to be resolved in future studies is the biological significance of the upregulation of ribosomal and mitochondrial transcripts in myeloid and other cell types observed here in familial tauopathy. For example, do these changes in gene expression in NC monocytes primarily reflect increased fragility and/or decreased survival of these cells in tauopathy, or are they reflective of more specific changes in ribosomal and mitochondrial biology?

As alluded to above, it will also be important to determine which peripheral immune changes are conserved between familial tauopathy and diverse forms of sporadic primary and secondary tauopathy. In addition, given the complex temporal trajectories of brain myeloid responses in tauopathy [107], future research on large cohorts of presymptomatic *MAPT* pathogenic variant carriers will be needed to determine which peripheral changes observed here occur prior to disease onset. Finally, it will be important to ascertain whether the peripheral leukocyte changes discovered here are reflected by parallel changes in brain myeloid cells in individuals with tauopathy.

## Conclusions

To our knowledge, this is the first scRNA-seq study of peripheral blood cells in primary tauopathy. Beyond our initial discoveries, we validated a handful of DEGs via an orthogonal technique, ddPCR. In particular, we have connected longstanding observations from mouse models regarding microglial *Cx3cr1* and tau neuropathology to reduced *CX3CR1* in peripheral leukocytes in individuals with familial tauopathy. Moreover, we discovered a significant reduction in the abundance of circulating NC monocytes, a cell type that is similarly reduced in several additional neurodegenerative diseases. We also discovered large numbers of DEGs in NK cells, including *CX3CR1*, which is thought to be involved in recruitment of NK cells to the CNS. Further studies are now required to investigate the generalizability of our findings through replication in larger cohorts and extension to other tauopathies and related neurodegenerative diseases. Analogous studies of PBMCs in *GRN* pathogenic variant carriers and *C9orf72* hexanucleotide repeat expansion carriers should enable the discovery of peripheral biomarkers of FTLD-TDP. Ultimately, comparative studies should clarify the role of peripheral immune responses in distinct proteinopathies and enable discovery of novel peripheral biomarkers that can successfully discriminate between tau and TDP-43 neuropathology, providing critical new tools for diagnostics and clinical trials.

## Supplementary Information

**Additional file 1: Table S1.** Cell Ranger metrics  summarizing important characteristics of the detected cells for each sample.

**Additional file 2: Table S2.** Cell counts during quality-control steps for each sample.

**Additional file 3: Table S3.** RNA integrity number for PBMC samples.

**Additional file 4: Figures S1-S9.** All supplementary figures.

**Additional file 5: Table S4.** Cell-type specific differential expression analyses.

## Data Availability

The scRNA-seq dataset described here has been uploaded to the FAIR Data Sharing Portal within the Alzheimer’s Disease Workbench, which is supported by the Alzheimer’s Disease Data Initiative, and can be accessed at https://fair.addi.ad-datainitiative.org/#/data/datasets/single_cell_rna_seq_data_derived_from_mapt_carriers_and_controls.

## Abbreviations

AD: Alzheimer’s disease
ALS: Amyotrophic lateral sclerosis
ALSP: Adult-onset leukoencephalopathy with axonal spheroids and pigmented glia
CAT: Center for Advanced Technology
cDC: Conventional dendritic cell
CDR-SB: Clinical Dementia Rating scale Sum of Boxes
CNS: Central nervous system
CSF: Cerebrospinal fluid
dd: Droplet digital
DEG: Differentially expressed gene
FDR: False discovery rate
FTD: Frontotemporal dementia
FTLD: Frontotemporal lobar degeneration
IHG: Institute for Human Genetics
LFC: Log_2_ fold change
LPS: Lipopolysaccharide
MAC: Memory and Aging Center
MCL: Markov cluster algorithm
NC: Nonclassical
NK: Natural killer
PBMCs: Peripheral blood mononuclear cells
PCA: Principal component analysis
PD: Parkinson’s disease
PET: Positron emission tomography
QC: Quality control
RIN: RNA integrity number
RT: Reverse transcription
scRNA-seq: Single-cell RNA sequencing
UCSF: University of California, San Francisco
UMAP: Uniform manifold approximation and projection
