# Supplementary material for: Single-cell RNA-seq reveals alterations in peripheral CX3CR1 and nonclassical monocytes in familial tauopathy
Source: Genome Med. 2023 Jul 18;15:53. doi: 10.1186/s13073-023-01205-3 (PMC10354988; doi:10.1186/s13073-023-01205-3)
Supplement: Supplementary file 4 — Additional file 4: Figures S1-S9. All supplementary figures. [file 13073_2023_1205_MOESM4_ESM.pdf]

Figure S1

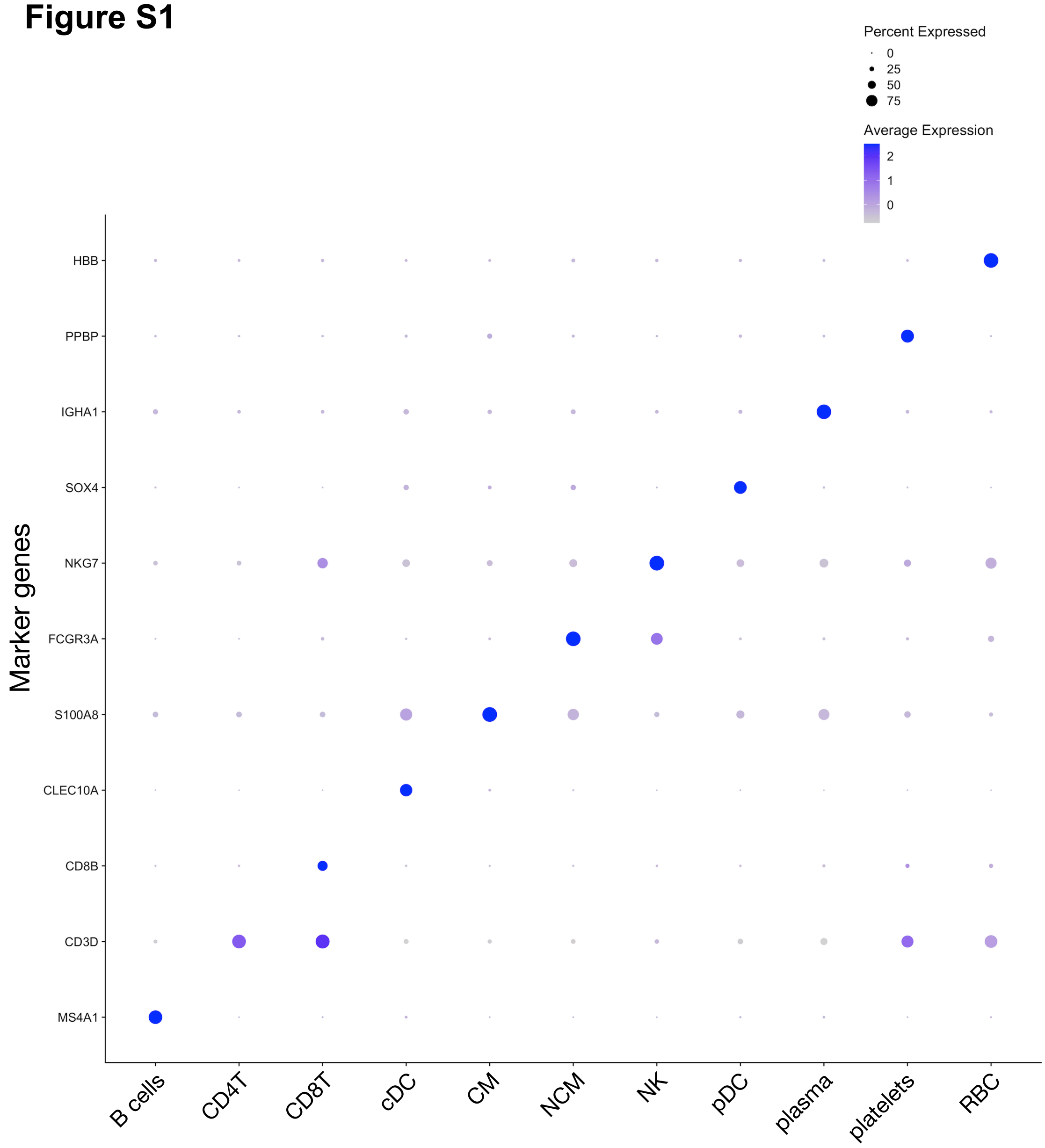

Figure S1. Annotation of cell types from cluster markers. Cell types were annotated using a combination of canonical marker genes and additional markers identified via Seurat's `FindMarkers` function. Clusters were assigned to the following cell-type categories: B cells (clusters 8, 12, 19), $CD4^{+}$ T cells (CD4T; clusters 0, 1, 7, 10, 17), $CD8^{+}$ T cells (CD8T; clusters 4, 5, 6, 9), cDC (cluster 14), classical monocytes (CM; cluster 2), NC monocytes (NCM; cluster 11), NK cells (clusters 3 and 15), plasmacytoid dendritic cells (pDC; cluster 16), plasma cells (cluster 18), and contaminating platelets (cluster 13) and red blood cells (RBC; cluster 20). Expression of marker genes is shown using Seurat's `DotPlot` visualization, with dot size indicating the percentage of cells in a given category having detectable expression of a given marker gene and color indicating the average expression level.

Figure S2

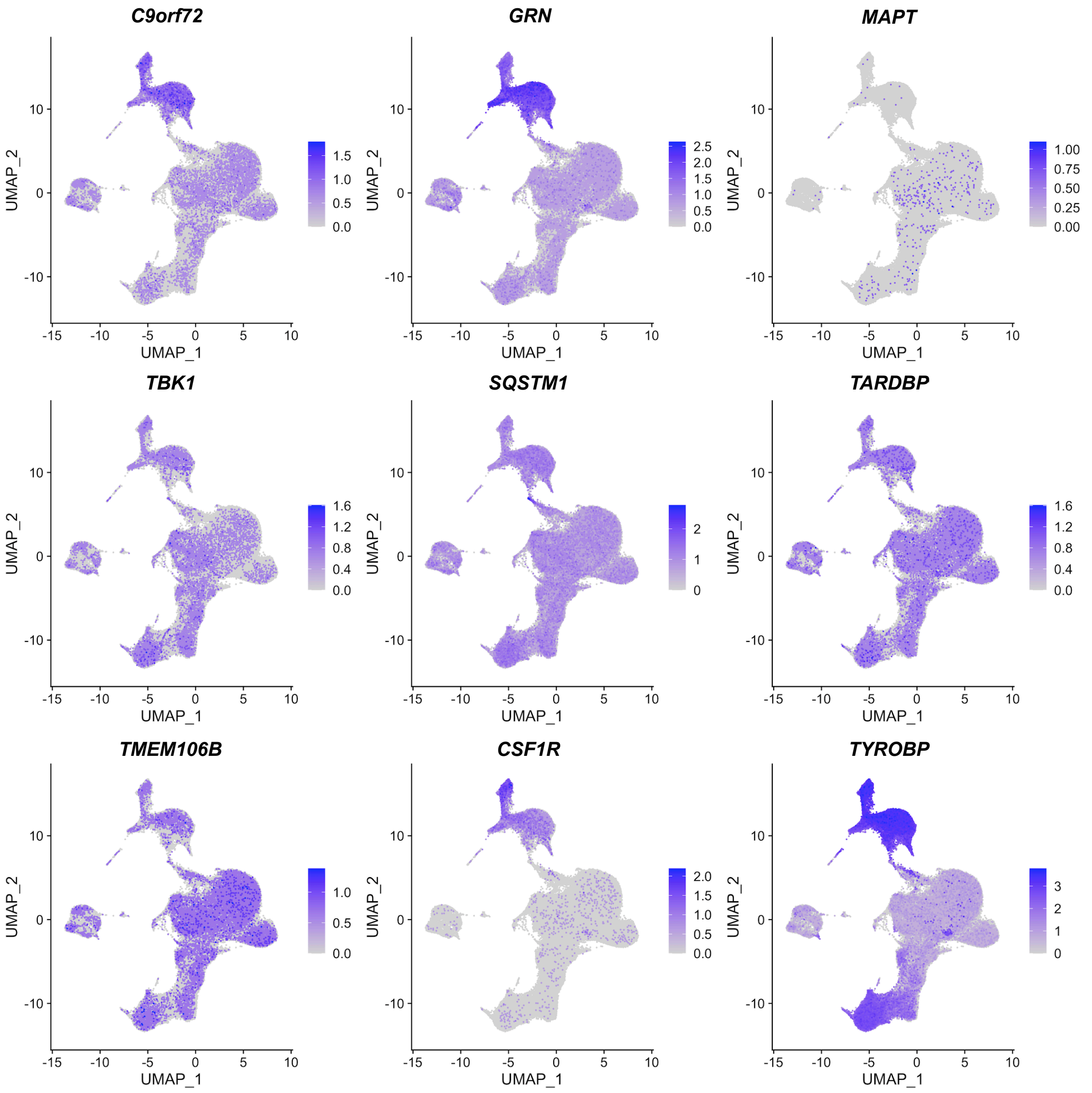

Figure S2. Expression of FTD-associated genes in PBMCs. The expression level of nine FTD-associated genes (*C9orf72*, *GRN*, *MAPT*, *TBK1*, *SQSTM1*, *TARDBP*, *TMEM106B*, *CSF1R*, *TYROBP*) across all PBMCs in the dataset is depicted. Some genes displayed widespread expression across many PBMC cell types (*TBK1*, *SQSTM1*, *TARDBP*, *TMEM106B*), while others showed enriched expression in myeloid clusters (*C9orf72*, *GRN*, *CSF1R*, *TYROBP*). In contrast to other FTD-associated genes, *MAPT* expression was very rarely detected in PBMCs.

Figure S3

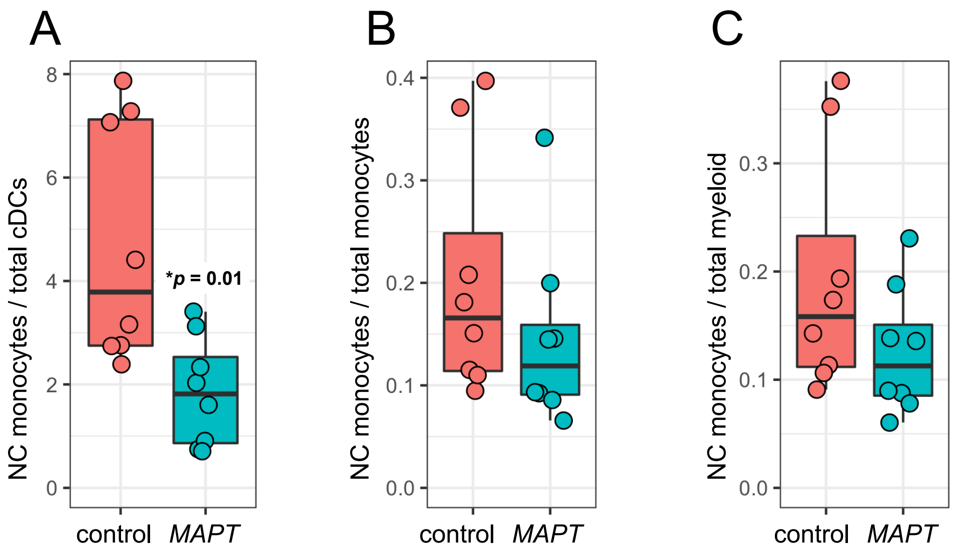

Figure S3. Nonclassical monocyte abundance relative to other myeloid populations. A *MAPT* pathogenic variant carriers showed a significant reduction (p = 0.01) in the ratio of NC monocytes to total cDCs (cDC1 + cDC2). **B**, **C** In contrast, the ratio of NC monocytes to total monocytes (**B**) or total myeloid cells (**C**) was not significantly reduced in *MAPT* variant carriers compared to non-carrier controls.

Figure S4

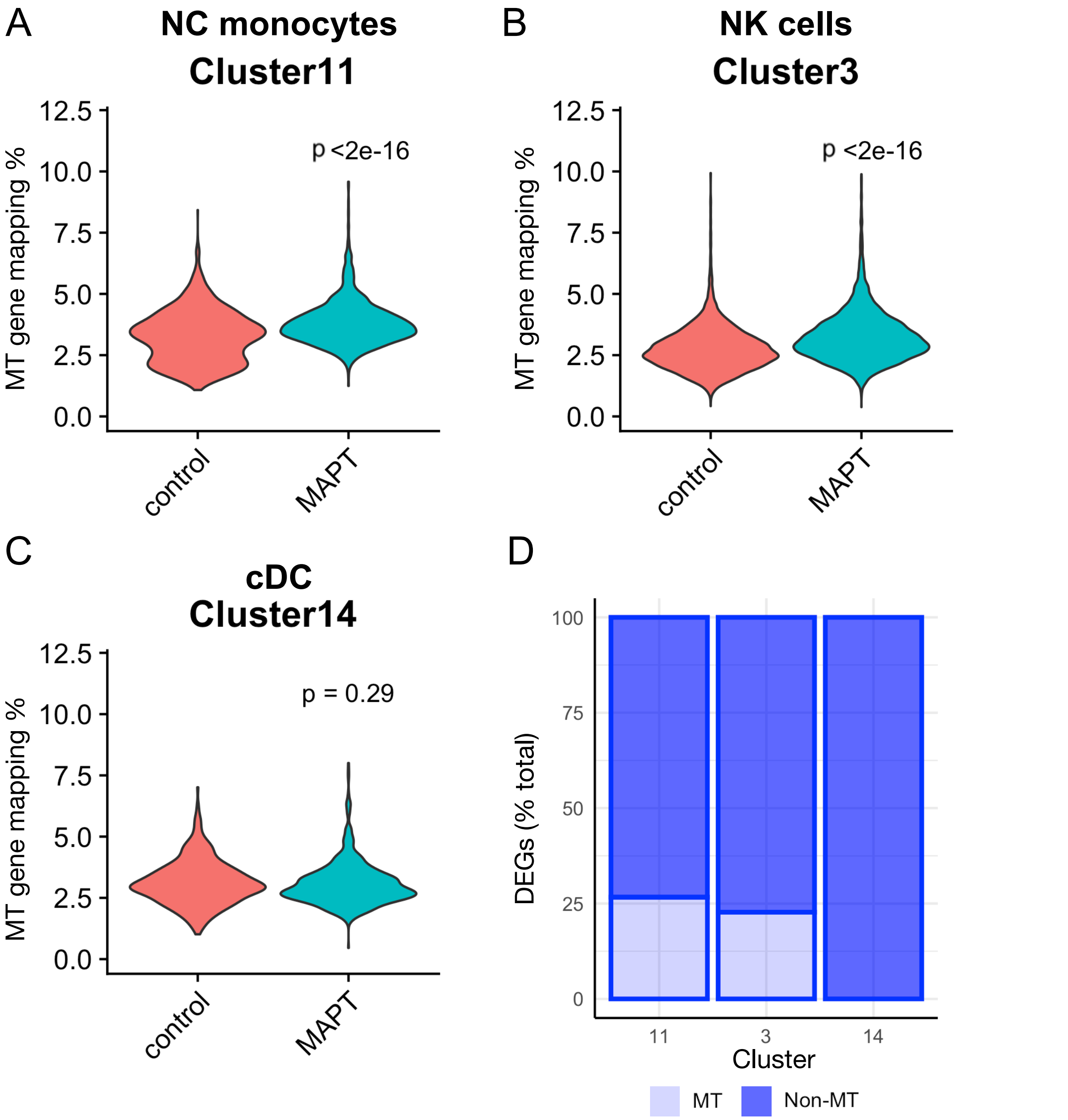

Figure S4. Mitochondrial genome mapping percentages and mitochondrial DEGs in selected clusters. A, **B** Mitochondrial mapping percentage was subtly but significantly increased ($p < 2 \times 10^{-16}$) in *MAPT* pathogenic variant carriers in clusters 11 (NC monocytes; **A**) and 3 (NK cells; **B**). **C** On the other hand, mitochondrial mapping percentage was not significantly increased in cluster 14 (cDC). **D** Clusters 11 and 3 both harbored appreciable fractions of mitochondrial DEGs relative to total DEGs, while cluster 14 did not contain mitochondrial DEGs.

Figure S5

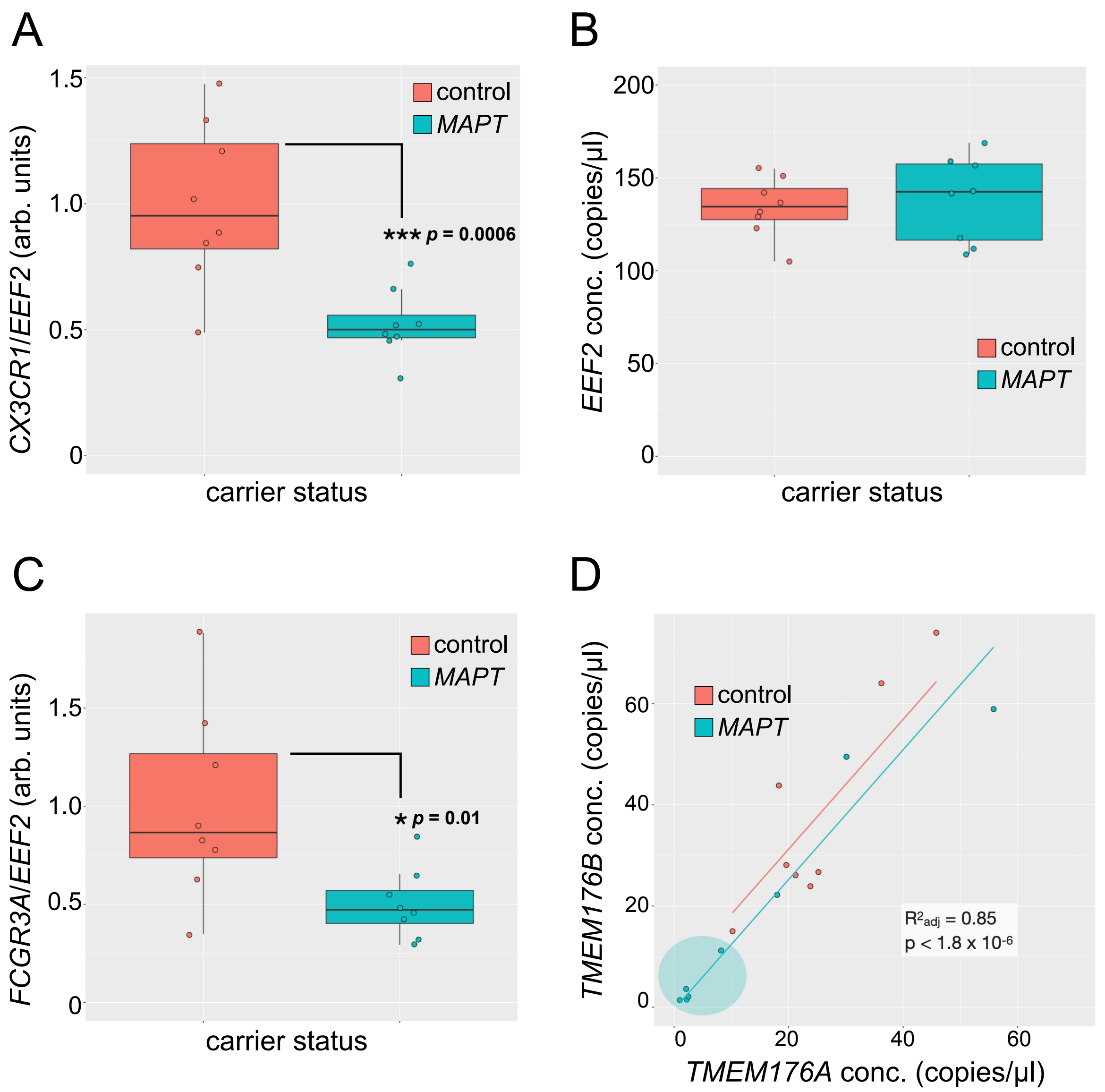

Figure S5. Additional droplet digital PCR analysis. A Normalization of the *CX3CR1* concentration to that of reference gene *EEF2* produced results similar (***, p = 0.0006) to what is depicted in Fig. 5A. **B** *EEF2* absolute concentration values were similar between *MAPT* pathogenic variant carriers and non-carrier controls. **C** Normalization of the *FCGR3A* concentration to that of *EEF2* did not alter the results depicted in Fig. 6B (*, p = 0.01). **D** *TMEM176A*/*B* levels were closely associated with one another (R^2_{adj} = 0.85, $p < 1.8 \times 10^{-6}$). A subset of *MAPT* variant carriers (5 of 8) showed levels of *TMEM176A*/*B* that were lower than what was observed for any non-carrier control.

Figure S6

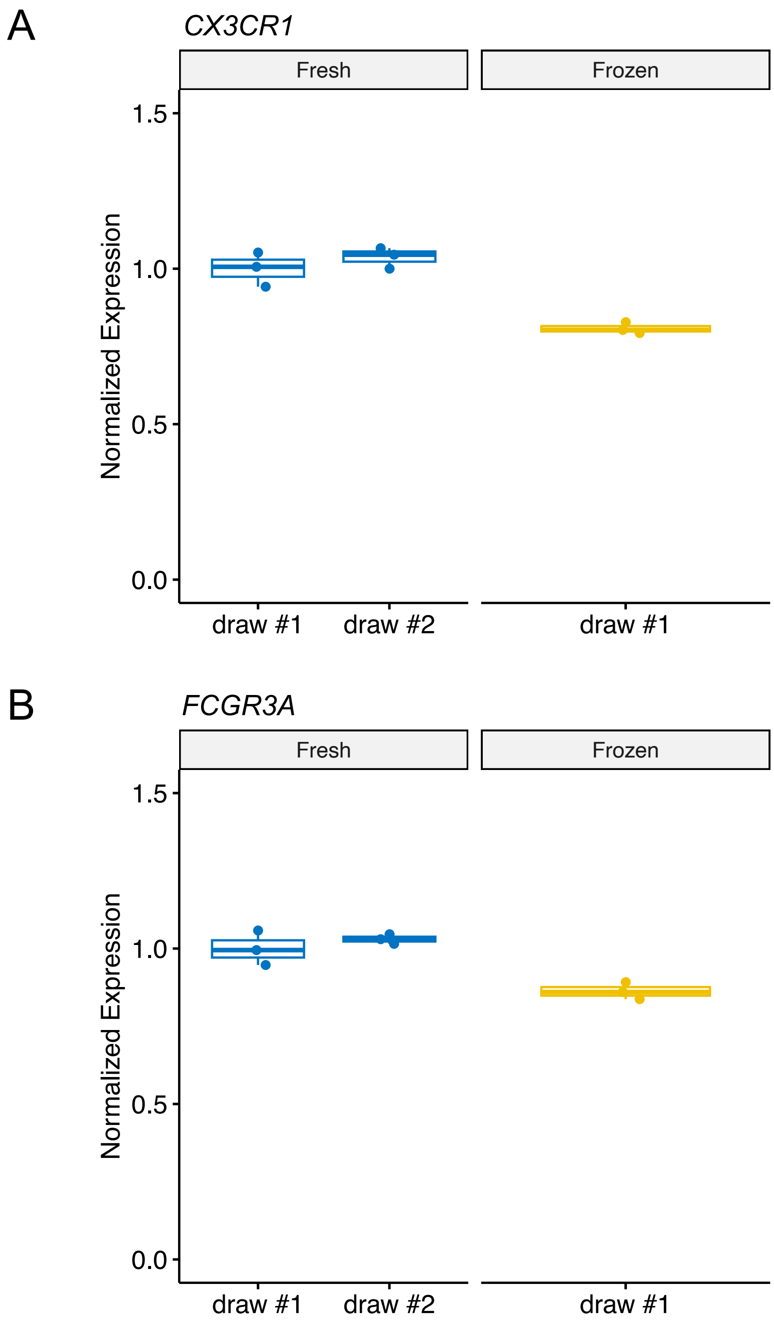

Figure S6. *CX3CR1* and *FCGR3A* expression in fresh vs. frozen PBMCs. *CX3CR1* (**A**) and *FCGR3A* (**B**) levels were measured together with reference gene *EEF2* by ddPCR using RNA derived from freshly isolated PBMCs (from two sequential blood draws from a research participant) and compared to RNA derived from cryopreserved and thawed PBMCs from the same participant. Both genes show ~15-20% reductions in expression in the cryopreserved PBMCs relative to the freshly isolated PBMCs.

Figure S7

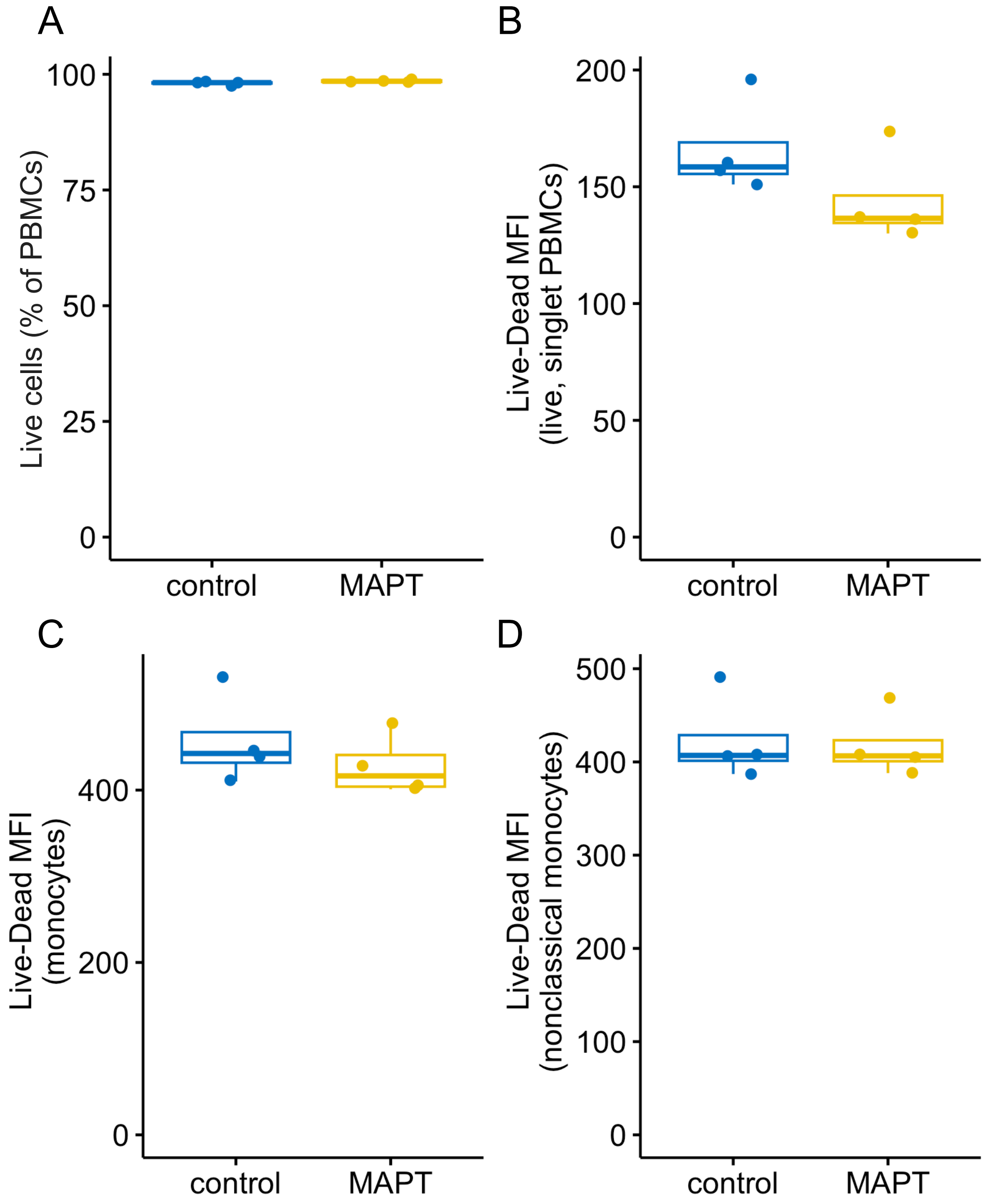

Figure S7. Viability of *MAPT* pathogenic variant carrier PBMCs. A The frequency of viable PBMCs (expressed as a percentage of all PBMCs), as defined by the viability gate (Fig. 9A), was >98% for *MAPT* pathogenic variant carriers and non-carrier controls. The LIVE/DEAD Aqua signal (MFI) within live, singlet PBMCs (**B**), monocytes (**C**), and nonclassical monocytes (**D**) was similar between *MAPT* variant carriers and non-carrier controls.

Figure S8

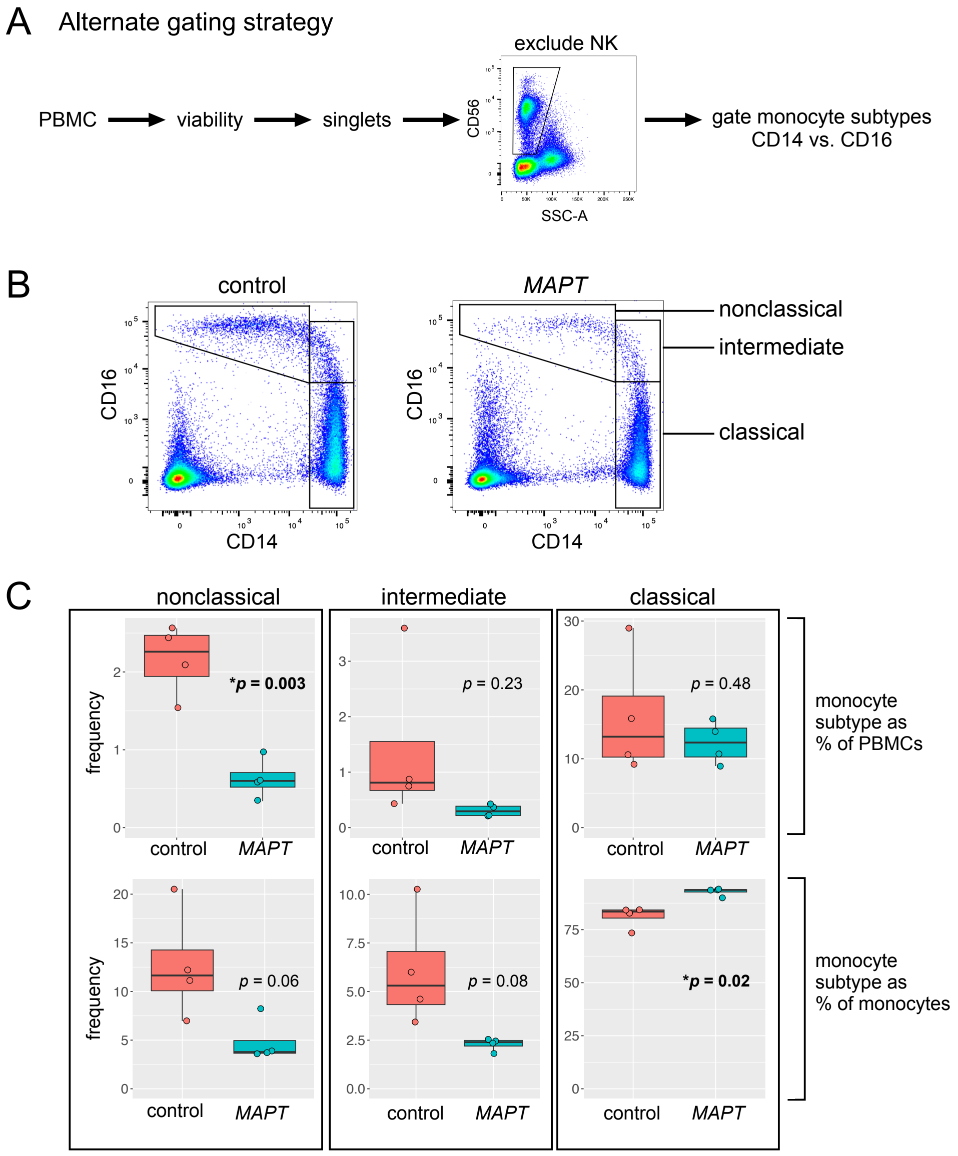

Figure S8. Alternate gating scheme for quantification of monocyte subtypes by flow cytometry. A Cells were gated as in the primary analysis for the first three gating steps. Next, $CD56^+$ NK cells were excluded, and monocyte subtypes were subsequently gated based on CD14 and CD16 expression. This gating scheme enables the selection of additional NC monocytes with lower levels of CD14 expression (**B**). **C** Quantification of the frequency of NC (left), intermediate (center), and classical monocytes (right), either as a percentage of PBMCs (top row) or all monocytes (bottom row). NC monocytes were reduced in *MAPT* pathogenic variant carriers as a fraction of PBMCs (upper left, $p = 0.003$). As a fraction of monocytes, NC monocytes again trended lower but did not reach significance (lower left, $p = 0.06$). Intermediate monocytes (center) showed a trend toward reduction relative to both PBMCs and monocytes. Classical monocytes (right) showed no change as a fraction of PBMCs but were significantly increased in *MAPT* pathogenic variant carriers as a fraction of all monocytes ($p = 0.02$).

Figure S9

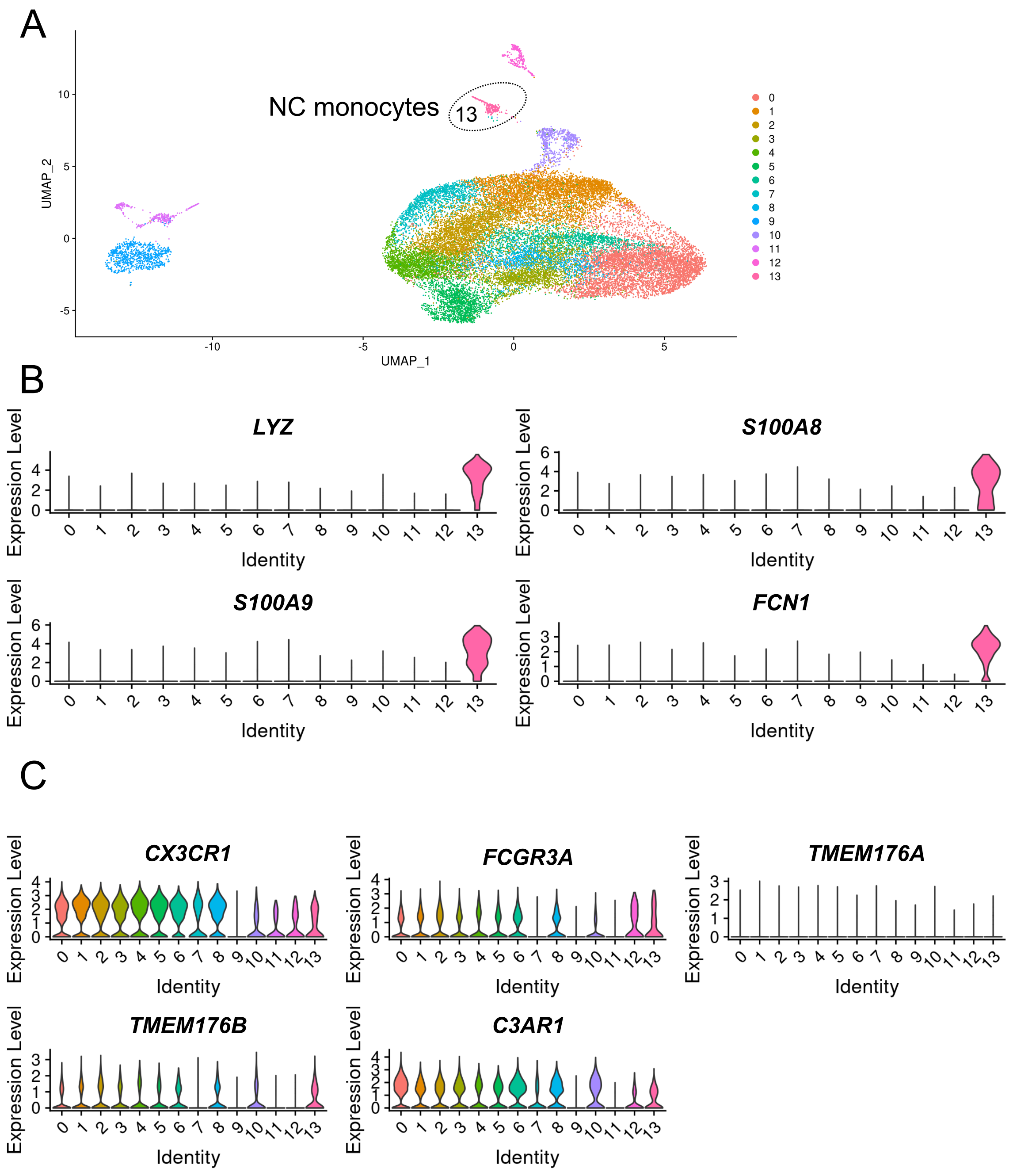

Figure S9. Analysis of gene expression in human brain nonclassical monocytes. Human brain scRNA-seq data originally from [46] were downloaded from GEO (accession GSE137444) and processed as described above for the PBMC scRNA-seq data. **A**, **B** CD16^{+} NC monocytes were identified in cluster 13 (**A**) and expressed marker genes *LYZ*, *S100A8*, *S100A9*, and *FCN1* (**B**; as described in [23]**). C** *CX3CR1*, *FCGR3A*, *TMEM176B*, and *C3AR1* showed robust expression in cluster 13 NC monocytes.
